# Immunoproteomic and Immunopeptidomic Analyses of *Histoplasma capsulatum* Reveal Promiscuous and Conserved Epitopes Among Fungi With Vaccine Potential

**DOI:** 10.3389/fimmu.2021.764501

**Published:** 2021-11-22

**Authors:** Brenda Kischkel, Camila Boniche-Alfaro, Isabela de Godoy Menezes, Suelen Andreia Rossi, Claudia Blanes Angeli, Sandro Rogério de Almeida, Giuseppe Palmisano, Leila Lopes-Bezerra, Joshua D. Nosanchuk, Carlos Pelleschi Taborda

**Affiliations:** ^1^ Department of Microbiology, Biomedical Sciences Institute, University of São Paulo, São Paulo, Brazil; ^2^ Department of Clinical and Toxicological Analysis, Faculty of Pharmaceutical Sciences, University of São Paulo, São Paulo, Brazil; ^3^ Department of Dermatology, Tropical Medicine Institute, Faculty of Medicine, University of São Paulo, São Paulo, Brazil; ^4^ Department of Parasitology, Biomedical Sciences Institute, University of São Paulo, São Paulo, Brazil; ^5^ Division of Infectious Diseases, Department of Medicine, Albert Einstein College of Medicine, New York, NY, United States; ^6^ Department of Microbiology and Immunology, Albert Einstein College of Medicine, New York, NY, United States

**Keywords:** vaccine, pan-fungal, peptide, dimorphic-fungi, proteomic

## Abstract

As there are more than 6 million human deaths due to mycoses each year, there is an urgent need to develop fungal vaccines. Moreover, given the similarities among pathogenic fungi, it may be possible to create a multi-fungi vaccine. In this study, we combined immunoproteomic and immunopeptidomic methods, for which we have adapted a technique based on co-immunoprecipitation (Co-IP) that made it possible to map *Histoplasma capsulatum* epitopes for the first time in a natural context using murine dendritic cells (DCs) and macrophages (Mφ). Although polysaccharide epitopes exist, this research focused on mapping protein epitopes as these are more immunogenic. We used different algorithms to screen proteins and peptides identified by two-dimensional electrophoresis (2-D) and Co-IP. Seventeen proteins were revealed by 2-D gels, and 45 and 24 peptides from distinct proteins were presented by DCs and Mφ, respectively. We then determined which epitopes were restricted to MHC-I and II from humans and mice and showed high promiscuity, but lacked identity with human proteins. The 4 most promising peptides were synthesized, and the peptides with and without incorporation into glucan particles induced CD4+ and CD8+ T cell proliferation and produced a Th1 and Th17 response marked by the secretion of high levels of IFN-γ, IL-17 and IL-2. These epitopes were from heat shock protein 60, enolase, and the ATP-dependent molecular chaperone HSC82, and they each have a high degree of identity with proteins expressed by other medically important pathogenic fungi. Thus, the epitopes described in this study have the potential for use in the development of vaccines that could result in cross-protection among fungal species.

## 1 Introduction

Invasive fungal infections remain a major public health problem and there are no licensed vaccines to combat any human mycosis. The global estimate of deaths from invasive fungal infections is 6 million per year, which emphasizes the importance of developing safer and more effective ways to prevent and treat these complex infections (1). Notably, the continued increase in numbers of immunocompromised individuals, such as transplant and implant recipients, people living with the human immunodeficiency virus (HIV), cancer patients, and individuals receiving immunosuppressants, has worried the scientific community, since these individuals are highly susceptible to and more frequently develop serious fungal infections (2).

The fungal cell wall and plasma membrane are composed of polysaccharides and a variety of proteins conserved between species. These conserved proteins can potentially be utilized to develop a single vaccine to protect against different types of mycoses (3). An example of a “pan-fungal” vaccine candidate was reported by Wuthrich et al. (4), demonstrating that calnexin, an conserved antigen present on the surface of the fungal cell and a 13-mer peptide derived from this protein induced the expansion of CD4^+^ T cells.

One of the biggest challenges in the development of vaccines or immunotherapies targeting fungal pathogens comes from the fact that these organisms are complex eukaryotic cells and many candidate proteins show some level of similarity to human proteins (5). For example, immunization with *H. capsulatum* recombinant heat shock protein 60 (rHsp60) provides protection against experimental infection. However, the application of rHsp60 as a vaccine is limited, since this protein has high identity with the human Hsp60, which can potentially lead an autoimmune reaction (6). This problem is also seen in classic vaccines that make use of microorganisms or whole proteins. These formulations can lead to allergenic and/or reactogenic responses when presenting an unnecessary antigenic load (7). In contrast, the development of bioinformatics tools has led to the identification of peptides in sequences of immunogenic proteins containing only the epitope recognized by T cells capable of inducing a specific and targeted immune response (7, 8). Based on predictive analyses, several studies have shown the effectiveness of vaccines against infectious diseases using peptides (4, 9–11).

Adaptive immunity plays a major role in resistance and is crucial for the protection and resolution of fungal infections. DCs and Mφ are antigen presenting cells (APCs) capable of internalizing and processing a fungal pathogen to generate antigenic peptides. These peptides bind stably to MHC-I and MHC-II molecules, and are presented on the cell surface and recognized by CD8^+^ and CD4^+^ T lymphocytes, respectively (12).

In the present study, we used a Co-IP strategy to capture *H. capsulatum* peptides coupled to DCs or Mφ MHC-II molecules and identified those peptide sequences naturally presented by these APCs after fungal phagocytosis. Cryptic species nested in the *H. capsulatum* complex cause histoplasmosis, an endemic mycosis with manifestations ranging from a mild flu-like illness to pneumonia to disseminated infection, which is frequently lethal (13). This fungus has genetic similarities with other clinically important dimorphic fungi such as *Paracoccidioides* species, *Blastomyces dermatitidis*, *Sporothrix* species and *Coccidioides* species (4). This fact led us to describe selected conserved protein sequences and/or related to antigens found in other fungal pathogens. Thus, pan-fungal and exclusive epitopes from *H. capsulatum* described in this study are putative targets for vaccine.

## 2 Material and Methods

### 2.1 Animal Use and Ethics Statement

In this study, male C57BL/6 mice (age 6 to 8 weeks) were used as this species is susceptible to infection by *H. capsulatum* (14). Mice were obtained from the Animal Facility at the University of São Paulo (USP) School of Medicine in pathogen-free conditions. This study is in accordance with Law 11.794 of October 8, 2008; Decree 6899 of July 15, 2009, as well as with the rules issued by the National Council for Control of Animal Experimentation (CONCEA). All murine work was approved by the Ethic Committee on Animal Use (number 1169061218) of the Biomedical Sciences Institute (CEUA-ICB/USP).

### 2.2 Strain and Culture Conditions

In this study, reference strain *H. capsulatum* (ATCC 26032; designation G-217B) was obtained from the American Type Culture Collection (Rockville, MD, EUA). *H. capsulatum* was cultivated in the yeast phase on Brain Heart Infusion (BHI - BD, Sparks, MD, USA) medium, supplemented with 1% (w/v) glucose (BD, Sparks, MD, USA), 1.5% (w/v) agar and 0.1% (w/v) L-cysteine (Sigma-Aldrich, St. Louis, MO, USA) at 37°C.

### 2.3 Immunoproteomic: Characterization of the Antigenic Profile

#### 2.3.1 Biomass Production and Protein Extraction

An inoculum of 5x10^6^ viable cells/ml of *H. capsulatum* was prepared in phosphate buffered saline (PBS) and counted in Neubauer’s chamber using Trypan blue dye (Sigma-Aldrich, UK) to measure viability. The inoculum was transferred to BHI liquid medium and incubated at 120 rpm for 7 days at 37°C. After incubation, the cells were centrifuged at 5000 rpm for 20 min and washed three times in ultrapure water. The pellets obtained were submitted to extraction with Tris-MgCl_2_ buffer pH 8.3, containing 0.5 M Tris-HCl, 2% (w/v) CHAPS, 20 mM MgCl_2_ (both Sigma-Aldrich, St. Louis, MO, USA), 2% (w/v) dithiothreitol (DTT – Invitrogen, CA, USA), protease inhibitor cocktail (complete EDTA-free, Roche, Mannheim, Germany) and 600-μm glass beads (1:1). These cells were vortexed for 10 min at 4°C and then, centrifuged at 5000 rpm for 20 min at 4°C. The supernatant was transferred for a new tube. Then, Tris-MgCl_2_ buffer was added and a new extraction were performed twice. Supernatants were aliquoted and kept until -80°C until use. The entire extraction procedure was performed in an ice bath. The protein concentration in the extract was measured using the Bradford method.

#### 2.3.2 Production of Polyclonal Antibodies

Two groups of seven C57BL/6 mice each were inoculated through the intratracheally route with 10^6^ cells/in 50 μl yeast cells or PBS. After 14 days, the animals were euthanized and the blood was collected by intracardiac puncture. The blood collected was subjected to an incubation of 37°C for 30 min, followed by 4°C for 40 min with centrifugation at 3000 xg for 15 min. The sera were kept at -80°C until use.

#### 2.3.3 Two-Dimensional Gel Electrophoresis (2-D SDS-PAGE) and Immunoblotting

The quality and reproducibility of the protein profile of each extract (biological replicates) was verified by 12% 1-D SDS-PAGE (15). For 2-D SDS-PAGE, 300 μg of proteins were precipitated by TCA/acetone according Niu et al. (16), and suspended in rehydration buffer, containing 7 M urea, 2 M thiourea, 4% (w/v) CHAPS, 1% (w/v) DTT, 2% (v/v) IPG buffer and bromophenol blue traces. The proteins were added onto immobilized pH gradient strips (pH 3-10, 13 cm, Immobiline DryStrip, GE Healthcare) and hydrated at 30 V for 12 h. The strips were placed in an Ettan IPGphor III and subjected to the following sequential steps: Step 30 V for 12h; Step 500 V for 500 V/h; Grad 1000V for 800 V/h; Grad 8000 V for 11300 V/h and Step 8000 V for 2900V/h. Next, the strips were reduced for 40 min with 0.5% DTT and alkylated for 40 min with 2.5% iodoacetamide diluted in equilibration buffer (6 M urea, Tris-HCl pH 8.8, 20% glycerol, 2% SDS and bromophenol blue traces. To create the second dimension, the strips were positioned between the glass plates under a 12% polyacrylamide gel and sealed with 0.5% agarose containing traces of blue bromophenol. The electrophoretic run was performed at 4°C, 150 V for 60 min and 250 V until the end of the run (approximately 4-5 hours) using a SE 600 Ruby Standard Dual Cooled Vertical Unit (GE Healthcare). A prestained molecular weight standard was used as a reference (Protein BenchMark, Invitrogen, Carlsbad, CA, USA). The proteins were stained with Coomassie blue or silver-stained and the images were captured by the ChemiDoc System (Bio-rad). For immunoblotting, the 1D and 2D gels were transferred to 0.45 µm nitrocellulose membranes using a *semi dry transfer cell* system (Trans-blot, Bio-Rad). Assays were then performed as described by Almeida et al. (17). Polyclonal mouse serum (1:100) collected from infected mice was used as primary antibody. Blots were imaged in a transilluminator (Bio-rad) and analyzed by ImageMaster 2D Plattinum software (Genebio, Geneva, Switzerland) version 6.0.

#### 2.3.4 Tryptic Digestion of Spots and Elution of Peptides

The spots revealed by the Western blot were excised from the SDS-PAGE Gel stained by coomassie blue, placed into Protein LoBind tubes (Eppendorf, Hamburg, Germany) with washing solution [50 mM ammonium bicarbonate (Ambic) in 40% acetonitrile (ACN)] and then shaken on a thermomixer at 1400 rpm. The fragments were dehydrated with 100% acetonitrile by vortexing and then hydrated with 10 mM DTT in 50 mM Ambic at 1400 rpm, 56°C for 45 min. Then, 55 mM iodoacetamide alkylation solution (IAA) in 50 mM Ambic was added and the fragments were incubated in the dark at RT for 30 min. Afterwards, the samples were washed with 50 mM Ambic followed by dehydration with 100% ACN. The fragments were digested with 200 ng trypsin (Trypsin sequencing grade, Roche) for 16 h at 37°C on thermomixer at 500 rpm. The trypsin action was interrupted by the addition of 10% formic acid (TFA) and the supernatant was transferred to a new tube. Then, extraction solution (40% ACN in 0.1% TFA) was added to the gel pieces and the samples were shaken on a thermomixer at 1400 rpm for 15 min at RT. The supernatant was added to the new tube. The samples were dried in a vacuum centrifuge and suspended in 0.1% TFA. The samples were desalted using C18 membrane disk (stage tips) (18) for analysis by mass spectrometry.

### 2.4 Immunopeptidomic: Purification of Peptides Naturally Presented by Macrophage and Dendritic Cells

#### 2.4.1 Bone Marrow Dendritic Cells and Macrophage Differentiation

Bone marrow from femurs and tibiae of C57BL/6 mice were obtained according to the protocol established by Inaba et al., (19). For differentiation in DCs, the cells were cultured in RPMI 1640 medium (LGC, biotechnology, Brazil) supplemented with 10% (v/v) fetal bovine serum (LGC, biotechnology, Brazil) 1% (v/v) penicillin/streptomycin (Gibco, USA), 30 ng/ml of GM-CSF and 15 ng/ml of IL-4 (Invitrogen, USA). The culture medium was changed on the third and fifth days. On day 8, differentiated dendritic cells were obtained. For differentiation in Mφ, the cells were cultured in R20/30 medium (RPMI 1640, 20% (v/v) fetal bovine serum and 30% (v/v) supernatant of L929 cells) (20). The medium was changed on the fourth day and, on the seventh day, macrophages were obtained. All cells were incubated in a humidified CO_2_ incubator at 37°C. The cell phenotype was confirmed by BD FACS Fortessa flow cytometer as described below.

#### 2.4.2 Phagocytosis Kinetics - Fluorescence Microscopy

Mφ and DCs were stimulated with 10 ng/ml IFN-γ for 20 h before the phagocytosis assay. *H. capsulatum* yeasts were inactivated at 56°C for 1 h and labeled with Calcofluor White Stain (sigma-Aldrich, St. Louis, MO, USA). Each well on the culture plate received a ratio of 3 yeasts to 1 cell. The phagocytosis periods of 0, 1, 2, 4, 6 and 8 h were evaluated. Lysosomal Staining Kit (Red fluorescence – Cytopainter, Abcam, UK) was used on the cultures following the manufacturer’s recommendations. Images were captured using an EVOS fluorescence microscope (Live Technologies, CA, USA). Giemsa staining was performed to count phagocytosed cells in a Nikon light electron microscope (eclipse E200 model, Japan). The number of yeast cells within approximately 50 macrophages per field were counted, and the percentage of phagocyted yeasts was determined.

#### 2.4.3 Immunophenotyping and Presentation Kinetics of MHC Class II

Flow cytometry was used for immunophenotyping cells before and after differentiation and to determine the time interval (1, 2, 4, 6 and 8 h) at which the greatest expression of MHC-II molecules occurs by the differentiated cells after *H. capsulatum* phagocytosis. First, the cells were blocked for nonspecific anti-CD16/CD32 binding. DCs from mice were labeled with the following antibodies: anti-CD11c (clone HL3), anti-MHC-II (clone M5/114.15.2), anti-CD86 (clone GL1), anti-CD80 (clone 16-10A1) and Live/Dead Viability dye (ThermoFisher scientific). Mφ from mice were marked with: anti-CD11b (clone M1/70), anti-MHC II (clone M5/114.15.2), anti-F4/80 (clone BM8), anti-CD86 (clone GL1), anti-CD80 (clone 16-10A1) and Live/Dead Viability dye. Afterwards, the cells were washed with FACS buffer [PBS with 2% (v/v) fetal bovine serum and 0.1% (w/v) sodium azide] and fixed with 1% paraformaldehyde. The samples were assessed in a BD FACS Fortessa flow cytometer (Becton Dickinson, Palo alto, CA, USA) and the results analyzed using flowJo 10 software (TreeStar).

#### 2.4.4 MHC Class II Co-Immunoprecipitation (Co-IP)

The Co-IP tests were performed as described by Purcell et al. (21), with modifications. *H. capsulatum* phagocytosis experiments with approximately 5x10^8^ dendritic cells and macrophages were performed separately as described above. After 4 h phagocytosis, the cell culture bottles were washed twice with PBS. The murine cells were then removed from the bottles and centrifuged at 400 xg. The resulting pellet was subjected to extraction with Tris buffer [50 mM Tris-HCl pH 7.6, 150 mM NaCl, 1% (v/v) igepal-CA630 and protease inhibitor cocktail] under slow agitation at 4°C for 2 h. The extract was ultracentrifuged at 100,000 xg for 1 h. The resulting supernatant was subjected to immunoprecipitation. The Superparamagnetic Dynabeads Kit with protein G (Invitrogen, Lithuania) was used. An IgG2a anti-mouse I-Ab (Clone KH74) was used for linkage to the beads. Four-hundred μl of Beads and 96 μg of antibodies diluted in PBS were added to Protein LoBind tubes and incubated on a rotating rack at RT for 1 h. Then, the tubes were placed on a magnetic rack and the PBS removed and the beads were suspended in Ab binding washing buffer. The buffer was removed and 1200 μl of sample containing the antigens was added. The beads + antigens were incubated for 1 h on a rotating rack at RT. After, the samples were washed 3 times with washing buffer. The peptides were separated from the beads by adding 100 μl of elution buffer for 5 min at RT on a rotating rack. The tubes were placed on the magnet and the eluted peptides were transferred to a new LoBind tube.

#### 2.4.5 Tryptic Digestion of Proteins in Solution

The pH of the Co-IP samples was measured by pH Test Strips 4.5 - 10.0 (Sigma-Aldrich, St. Louis, MO, USA) and adjusted to pH 7 with 100 mM Ambic. The protein concentration in the sample was quantified by the Qubit Protein Assay Kit (Invitrogen, Oregon, USA). DTT was added at a final concentration of 10 mM and incubated for 45 min at 30°C, followed by 40 mM IAA and incubated for 30 min at RT in the dark. Then, 5 mM DTT was added and the samples were incubated for 15 min at 30°C. The pH was again measured (between 7 and 8) and 0.2 μg trypsin was added and the samples were incubated for 16 h at 37°C. The action of trypsin was stopped by the addition of TFA at the final concentration of 1%. The pH was measured (in pH 3). The samples were desalted using C18 membrane disk (stage tips) for analysis by mass spectrometry.

#### 2.4.6 Nanoflow Liquid Chromatography Coupled to LTQ-Orbitrap Velos MS and Data Analysis

Mass spectrometry (MS) analysis was performed at the BIOMASS Core Facility for Scientific Research of University of São Paulo, Brazil (CEFAP-USP) and was carried out on an LTQ-Orbitrap Velos ETD coupled with Easy NanoLC II (Thermo Scientific, Waltham, Massachusetts, USA) (22). After acquisition, the MS/MS spectra were searched against the UniProt *H. capsulatum* Protein Database (downloaded march, 2020). For immunoprecipitation, protein databases were generated for *H. capsulatum* and *Mus musculus*. The data were processing with MaxQuant software version 1.5.8.3 with the following parameters: trypsin enzyme (specific and semi-specific free N-terminus), initial precursor (MS) mass tolerance was 20 ppm in the first search and 4.5 ppm in the main search with fragment (MS/MS) mass deviation of 0,5 Da. Carbamidomethylation (Cys) (57.021464 Da) was used as the fixed modification, with two missed cleavages for trypsin. Oxidation on methionine (15.9949 Da), protein N-terminal acetylation (42.010 Da) were set as variable modifications. Proteins and peptides were accepted at a false-discovery rate (FDR) of less than 1%.

### 2.5 Bioinformatic Analyses and T-Cell Epitope Mapping

Information on protein functions was collected from the UniProt database. ProtParam software (https://web.expasy.org/protparam/) was used to determine theoretical isoelectric point (pI), instability index (II) and grand average of hydropathicity (GRAVY). The GRAVY value was calculated by adding the hydropathy values according to Kyte and Doolittle (23) in which hydrophobicity scores below 0 are more likely to be hydrophilic (globular) protein, while scores above 0 are more likely to be hydrophobic (fibrous) protein. Venn diagrams were generated by InteractiVenn software (http://www.interactivenn.net/). Basic Local alignment Search Tool (BLAST-p algorithm -(https://blast.ncbi.nlm.nih.gov/BlastAlign.cgi) was used to compare the proteins of *H. capsulatum* against *Homo sapiens* (taxid:9606) and other pathogens with genomes annotated in the NCBI database. The BLAST was made for *P. brasiliensis* (taxid:121759), *B. dermatitidis* (taxid:5039), *C. immitis* (taxid:5501), *S. schenckii* (taxid:29908), *A. fumigatus* (taxid:746128), *C. neoformans* (taxid:5207), *C. albicans* (taxid:5476), *C. auris* (taxid:498019) and proteins deposited in the Uniprot database. R software (https://cran.r-project.org/) was used to plot the heat map of the results obtained. The I-TASSER server (https://zhanglab.ccmb.med.umich.edu/I-TASSER/) was used for three-dimensional (3-D) structure modeling (24). PyMOL software (https://pymol.org/2/) was used to visualize 3-D structures and to highlight the sequence of peptides predicted as T cell epitopes in proteins. The refinement of the tertiary structure was carried out using the GalaxyRefine server (http://galaxy.seoklab.org/). The validation of the tertiary structure was performed through the generation of Ramachandran plots using the PROCHECK server (https://servicesn.mbi.ucla.edu/PROCHECK/).

#### 2.5.1 MHC Class I Binding Epitope Prediction

The NetMHC 4.0 server (https://services.healthtech.dtu.dk/service.php?NetMHC-4.0) was used for the identification of HLA-I-binding epitopes from the sequences of the whole proteins (25). In this study, 9-10-mer long peptides were predicted. For humans, the epitopes were predicted for binding affinity to 70 different HLA molecules, 36 HLA-A and 34 HLA-B. For mouse, the epitopes were predicted for 8 different H-2 allele, being H-2-Db, H-2-Kb, H-2-Dd, H-2-Kd, H-2-Kk, H-2-Ld, H-2-Qa1 and H-2-Qa2. The results are obtained in percentage (%) rank, in which peptides identified as strong binders (SB) are defined as having a rating of < 0.5, while weak binders (WB) are defined as having a rating of < 2. At the end, the generated SB peptides were compared with a sequence of human proteins using ClustalX 2.1 software and BLASTp to exclude identical peptides.

#### 2.5.2 MHC Class II Binding Epitope Prediction

The NetMHCII 2.2 Server (http://www.cbs.dtu.dk/services/NetMHCII-2.2/) was used for the identification of HLA-I-binding epitopes from the sequences of the whole proteins (26). Fifteen-mer long peptides were predicted. For humans, the epitopes was predicted for binding affinity to 14 HLA-DR alleles covering the 9 HLA-DR supertypes (HLA-DRB10101, HLA-DRB10301, HLA-DRB10401, HLA-DRB10404, HLA-DRB10405, HLA-DRB10701, HLA-DRB10802, HLA-DRB10901, HLA-DRB11101, HLA-DRB11302, HLA-DRB11501, HLA-DRB30101, HLA-DRB40101, HLA-DRB50101), 6 HLA-DQ (HLA-DQA10501-DQB10301, HLA-DQA10501-DQB10201, HLA-DQA10401-DQB10402, HLA-DQA10301-DQB10302, HLA-DQA10102-DQB10602, HLA-DQA10101-DQB10501) and 6 HLA-DP (HLA-DPA10103-DPB10401, HLA-DPA10103-DPB10201, HLA-DPA10201-DPB10101, HLA-DPA10201-DPB10501, HLA-DPA10103-HLA-DPB10301_DPB10401, and HLA-DPA10301-DPB10402). For mouse, the epitopes were predicted for 2 different allele H-2-IA, being H-2-IAb, H-2-IAd. The results were obtained in IC50 nM values. IC50 values less than 50 nm indicate strong binders (SB), values less than 500 nm indicate intermediate binders (IB), and values less than 5000 nm indicate weak binders (WB). The generated SB peptides and the peptides presented by macrophages and DCs were compared with a sequence of human proteins using ClustalX 2.1 software and BLASTp to exclude identical peptides.

#### 2.5.3 Prediction of Immunogenicity and IFN-γ Induction

The Class I Immunogenicity software (http://tools.iedb.org/immunogenicity/) was used to predict the immunogenicity of the class I peptide MHC complex. The prediction of immunogenicity for MHC-II was performed for sequences with 15 peptides using the CD4^+^ T cell immunogenicity prediction server (http://tools.iedb.org/CD4episcore/). The IFNepitope server (http://crdd.osdd.net/raghava/ifnepitope/) was used to predict IFN-γ induction by MHC-II binding peptides. The approach used for prediction was Motif and SVM hybrid.

### 2.6 Peptides

#### 2.6.1 Synthesis

Four peptides were synthesized by GenOne: FENLGARLL (HSP60); KPYVLPVPF (enolase); ILGDIGILTNATVFT (HSP60) and FAERIHKLVSLGLNI (HSC82). The 9 amino acid peptides had a neutral charge and the stock solutions were diluted in 20% methanol and 80% deionized H_2_O. The 15 amino acid peptides had acidic and basic charges and were diluted in 20% DMSO and 80% deionized H2O.

#### 2.6.2 Extraction and Synthesis of GPs

GPs was purified from baker’s yeast using a series of alkaline and acidic extraction steps as described (27). Five mg of dry GPs were swollen in 100 µg of diluted peptides and incubated for 2 hours at 4°C to allow diffusion of the peptides into the hollow GP cavity. In the case of GPs with more than one peptide inside, an equal amount of each peptide was added up to the total limit of 100 µg (e.g. for 2 peptides in the GP, 50 µg of each was added). The particles were frozen at -80°C and lyophilized. For the phagocytosis assay, the GPs were marked with FITC (1 mg/ml, fluorescein isothiocyanate isomer I, Sigma, St. Louis – MO, USA) for 30 min followed by repeated washes to remove residual dye.

#### 2.6.3 Cytotoxicity Assay

The safety of the peptides was evaluated by cytotoxicity assay using the colorimetric reagent MTT (3-(4,5-dimethylthiazol-2-yl)-2,5-diphenyltetrazolium bromide; Invitrogen, USA) as the manufacturer recommends. JawsII cells (ATCC^®^ CRL-11904™, immature dendritic cell; monocyte - bone marrow from C57BL/6) were used. Untreated controls, or wells containing only the peptide diluents or cell culture medium were used (28). Peptides (HcP 1-4) were evaluated at a concentration of 10 µg per well, GP-HcP 1-4 and GP-HcP 2,3,4; 2,3 or 1,4 were tested at 5 µg per well. The effects of treatments on the cells were observed at 24 and 48 hours. The assay was performed as described by Kischkel et al. (29).

#### 2.6.4 Spleen Cell Re-Stimulation

Spleens were harvested from mice on day 14 after infection and a single cell suspension was prepared using a 70 µm cell strainer. Cells were washed with RPMI and incubated in a red cell lysis buffer for 4 minutes. The cells were labeled with carboxyfluorescein succinimidyl ester (Invitrogen CellTrace CFSE Kit) according to the manufacturer’s instructions. Cells were centrifuged and suspended in cell culture media (RPMI 1640, 10% FBS, 1% penicillin/streptomycin, 20 mM HEPES, 1% sodium pyruvate, 1% MEM non-essential amino acids and 50 µM de 2-mercaptoethanol). Cells were adjusted at a density of 4x10^6^ cells/mL and 50 µL of the suspension were plated. 150 µL of media alone, or containing 10 µg HcP 1-4, 5 µg GP-HcP 1-4, 5 µg GP-HcP 2,3,4; 2,3 or 1,4, 5 µg GP empty or 2 µL phytohemagglutinin (PHA), were added to the cells. The plates were incubated at 37°C for 96 hours. Afterwards, the cell supernatants were removed to measure cytokine levels and the cells were stained with anti-CD3 (APC-Cy7), anti-CD4 (PE), anti-CD8 (PerCp) antibodies and evaluated by flow cytometry.

#### 2.6.5 Cytokine Measurements

Cytokine levels were measured with an ELISA assay according to the manufacturer’s protocol. The cytokines assayed were IFN-γ, IL-4, IL-2 (BioLegend, San Diego, CA, USA) and IL-17A/F (LEGEND MAX, BioLegend, San Diego, CA, USA).

### 2.7 Statistical Analyses

The software OriginPro 8.0 (OriginLab Corporation, Northampton, USA) was used for statistical analysis. Data were statistically evaluated by one-way analysis of variance (ANOVA) and *post hoc* comparisons of the means of individual groups were performed using Tukey’s test. Values of *p* ≤ 0.05 were considered statistically significant. Flow cytometry results were analyzed by FlowJo 10 software (TreeStar).

## 3 Results

### 3.1 Immunoproteomic: 2-D Electrophoresis Reveals Antigenic Proteins of *H. capsulatum*

The first step of our study was to determine possible antigenic targets in *H. capsulatum*. For this, we identified antigenic proteins through 2-D gel immunoblotting and peptides by co-immunoprecipitation (Co-IP). Both approaches provided a broad overview of candidate proteins for vaccine development.

**Figure 1:**
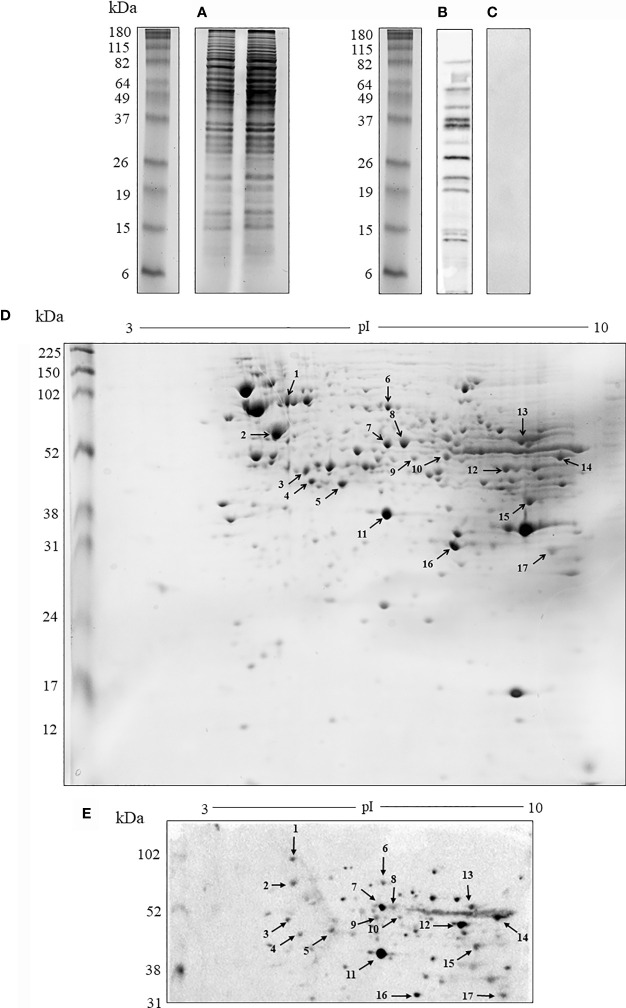
Analysis of the protein and antigenic profile of (*H*) *capsulatum*. **(A)** Electrophoretic profile by 1-D SDS-PAGE of protein extract. **(B)** Antigenic profile by immunoblotting the protein extract resolved in 1-D gel with sera from mice immunized with the protein extract. **(C)** 1-D gel control with serum from non-immunized animals. **(D)** Electrophoretic profile by 2-D SDS-PAGE of protein extract. The arrows indicate spots that have been excised and subjected to mass spectrometry. **(E)** Antigenic profile by immunoblotting of the protein extract with sera from mice immunized with the protein extract resolved in 2-D gel. The 1-D SDS-PAGE was also performed before each 2-D SDS-PAGE experiment to ensure the quality of protein extracts. The results are representative of the biological triplicates. Electrophoresis stained with Coomassie blue. kDa, kilodaltons.

*H. capsulatum* yeast proteins were obtained with a yield of approximately 3.4 mg/mL per extraction. The protein extract profile was evaluated by one-dimensional gel (1-D SDS-PAGE) showing proteins of molecular mass distributed between 5 and 180 kDa with a higher protein abundance between 30 and 115 kDa (**Figure 1A**). An immunoblot with sera from normal untreated mice did not show cross-reactivity of natural antibodies with these proteins (**Figure 1C**). On the other hand, the serum obtained from mice immunized with the protein extracts bound a wide range of antigenic proteins varying from 12 to 100 kDa of MW (**Figure 1B**). As expected, the protein profile obtained by two-dimensional gel (2-D SDS-PAGE) (**Figure 1D**) was abundant in a range similar to the 1-D gels. The 2-D immunoblot with sera of immunized animals (**Figure 1E**) showed about 41 protein reactive spots. Of these, 17 spots were excised from a Coomassie blue stained gel for further identification (spots set in **Figure 1D**). The protein spots were trypsinized and sequenced by nanoLC-MS/MS. **Table 1** shows the identified antigenic proteins.

**Table 1:** *H. capsulatum* antigenic proteins resolved by 2-D SDS-PAGE immunoblotting and identified by nanoLC-MS/MS sequencing.

Spot	Protein name	Accession number^a^
**1**	Heat shock protein SSC1	HCBG_08743
**2**	Hsp60-like protein	HCBG_08832
**3**	4-Hydroxyphenylpyruvate dioxygenase	HCBG_03027
**4**	Aha1_N domain-containing protein	HCEG_02400
**5**	Hsp70-like protein	HCBG_07920
**6**	Succinate dehydrogenase [ubiquinone] flavoprotein subunit	HCBG_06673
**7**	Homogentisate 1,2-dioxygenase	HCAG_05721
**8**	Aldehyde dehydrogenase	HCAG_08367
**9**	6-Phosphogluconate dehydrogenase	HCEG_08718
**10**	Enolase	HCEG_02034
**11**	Fructose-bisphosphate aldolase	HCBG_06745
**12**	Elongation factor 1-gamma	HCBG_08684
**13**	ATP synthase subunit alpha	HCBG_01891
**14**	Citrate synthase	HCEG_09064
**15**	Alcohol dehydrogenase	HCEG_08061
**16**	Peroxidase	HCDG_01107
**17**	Malate dehydrogenase	HCEG_05915

a Access number to database.

Certain characteristics of the whole proteins need to be considered in selecting the best vaccine candidates. Stability and hydrophobicity are important characteristics that may influence the heterologous expression of these targets in the future, as well as solubility can influence the candidate’s recognition by the APCs (30). In this study, they were decisive in our analysis to select the proteins that we would subject to the other prediction steps. Thus, stability, hydrophobicity and other characteristics of identified proteins such as function, molecular mass and pI were predicted and the results are shown in **Table S1**.

### 3.2 Immunopeptidomic: Bone Marrow Derived Mφ (BMDMs) and DCs (BMDCs) Phagocytose *H. capsulatum* Yeasts Within 4 h With High Expression of MHC-II

**Figure 2:**
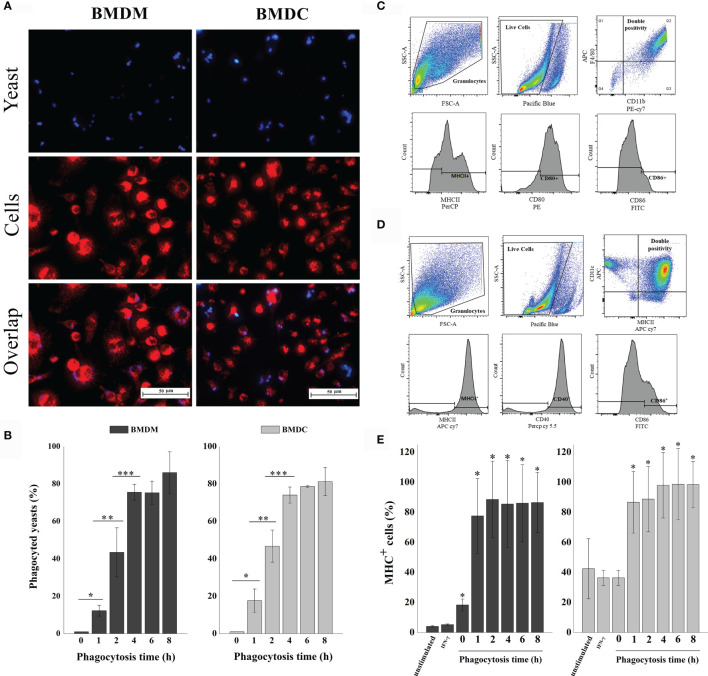
Phagocytosis of *H. capsulatum* yeasts by murine BMDM and BMDCs. **(A)** Fluorescence microscopy of yeasts (blue) ingested by BMDM or BMDC (red). Overlap: Both images, with yeast and BMDM or BMDC, represent the same quadrant of the culture plate well according to the phagocytosis time determined of 4 hours. **(B)** Determination of the percentage of phagocyted cells. The percentage of cells ingested was determined by counting the samples stained with giemsa under a light microscope. *differ from zero time, **differ from one time, ***differ from two time by the Tukey’s test, p < 0.01. **(C)** Analysis strategy used for phenotyping and activation of BMDM. First, the cells were separated by granularity (SSC) and size (FSC), followed by live/dead^-^ cell staining. Double positive cells for CD11b and F4/80 were classified as Mφ and analyzed for CD80, CD86 and MHC-II expression. **(D)** Analysis strategy used for phenotyping and activation of BMDCs. The cells were separated by granularity (SSC) and size (FSC), followed by live/dead^-^ cell staining. Double positive cells for CD11c and MHC-II were classified as DCs and analyzed for CD40 and CD86 expression. **(E)** Percentage of MHC-II expression by BMDM or BMDC. Unstimulated cells, only stimulated with IFN-γ and phagocytosis times of 0, 1, 2, 4, 6 and 8 hours. Unmarked, dead, and unstimulated cells were used as biological controls. *Differ from unstimulated control by the Tukey’s test, p<0.01. These results are representative of the biological triplicate.

We first determined the phagocytosis time of *H. capsulatum* heat-killed yeasts by BMDM and BMDCs. Lysosomal and calcofluor white staining were used to visualize phagolysosomes of mammalian cells and the endocytosed yeasts, respectively. A representative image of the 4-hour phagocytosis time is shown in **Figure 2A**. Cells were stained with Giemsa to count the number of ingested yeasts (**Figure 2B** **)**. Our results demonstrate that phagocytosis occurs in a time-dependent manner. Cell maturation is necessary for optimal presentation of antigens to T lymphocytes. The upregulation of co-stimulatory molecules such as CD40, CD80 CD86 and MHC-II are involved in this process (31). **Figures 2C, D** demonstrate that the cells were positively regulated during phagocytosis and the expression of MHC-II by both cells increased after 1 hour of interaction with the yeast cells and the levels remained stable up to 8 hours (**Figure 2E**).

### 3.3 Immunopeptidomic Identification of *H. capsulatum* Peptides That Are Naturally Processed and Presented by BMDM and BMDC

**Figure 3:**
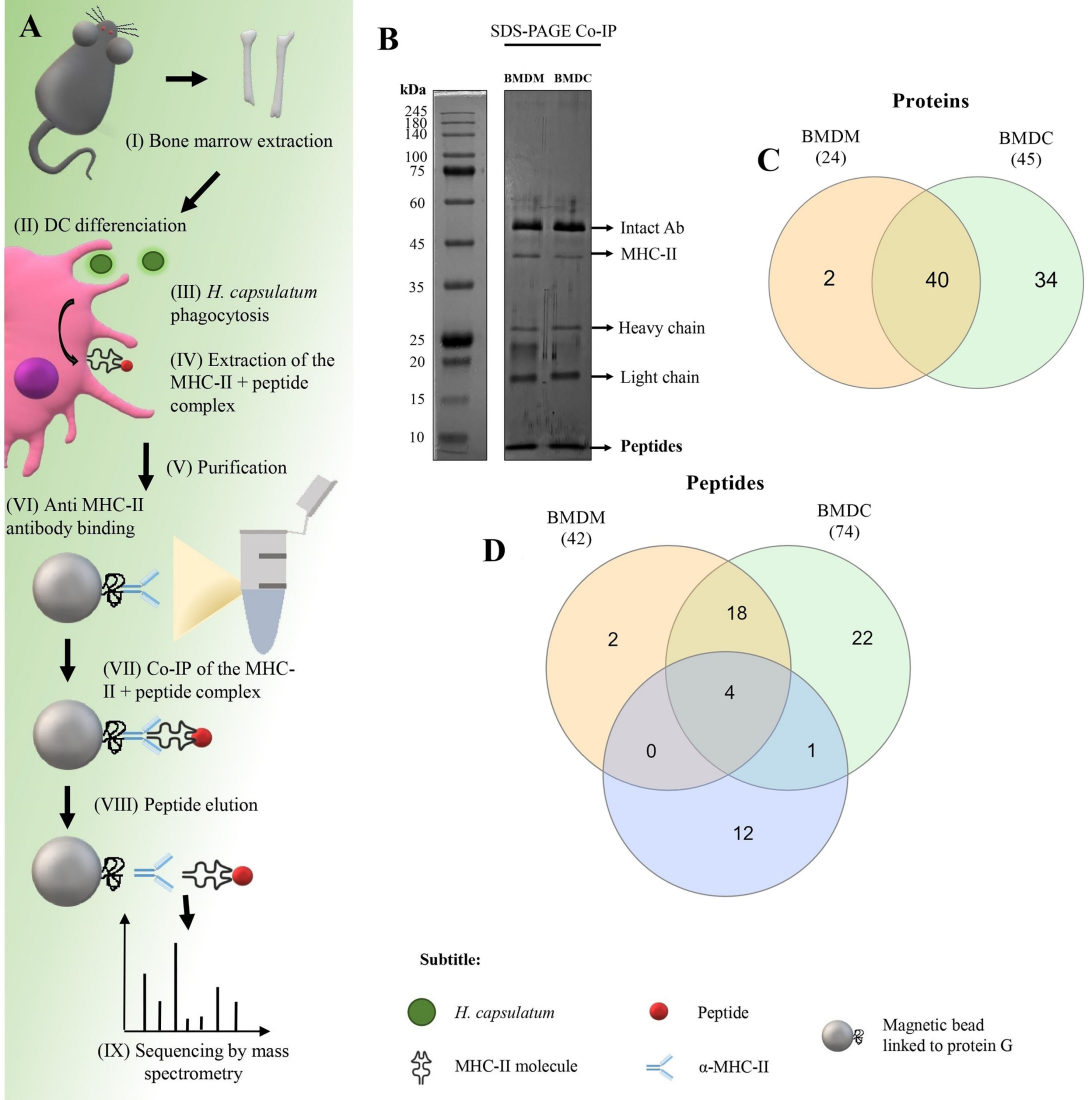
Co-immunoprecipitation (Co-IP) of *H. capsulatum* peptides linked to MHC-II from BMDM and BMDC. **(A)** Schematic representation of Co-IP. (I and II) Mφ and DCs were differentiated from bone marrow cells extracted from C57BL/6 mice. (III) After 4 hours of co-culture of murine cells with heat-killed yeast cells, (IV) MHC-II extraction was performed. (V and VI) Magnetic beads containing G protein were complexed with anti-mouse IgG2a I-Ab. After the incubation period, the amount of antibody in the supernatant was measured and total binding of antibody to magnetic beads was achieved. (VII) The extract was incubated for affinity capture of the MHC-II + peptide complexes. (VII) Then the peptides were dissociated from the column after acid denaturation and (IX) the eluted peptides were identified by nanoLC-MS/MS. **(B)** 1-D SDS-PAGE of the eluted peptide sample. Electrophoresis gels were stained with silver nitrate. kDa: kilodaltons. **(C)** Venn diagram showing common and exclusive proteins obtained through 2-D gel electrophoresis and Co-IP of *H. capsulatum* peptides linked to MHC II from BMDM and BMDCs. Orange ellipse: Proteins presented by BMDM. Green ellipse: Proteins presented by DMDC. Blue ellipse: proteins sequenced from 2-D gel extraction. Crossing the ellipses represents the proteins that were identified in common among Co-IP (BMDM and BMDCs) and 2-D gel electrophoresis. **(D)** Venn diagram showing common and exclusive peptides obtained Co-IP of *H. capsulatum* peptides linked to MHC II from BMDM and BMDCs. The results represent peptides sequenced by nanoLC-MS/MS. Orange ellipse: peptides presented by BMDM. Green ellipse: peptides presented by DMDC. Crossing the ellipses represents the peptides that were identified in common among BMDM and BMDCs.

The isolation and identification protocol for MHC-II-linked peptides used in this study is presented schematically in **Figure 3A**. After binding the MHC-II + peptide complex to beads, the peptides were eluted and an aliquot of the samples was separated by 1-D SDS-PAGE (**Figure 3B**) and another aliquot was used for sequencing by nanoLC-MS/MS. Approximately 121 µg/mls of peptides were eluted from both macrophages and DC. In **Figure 3B** the peptide band was observed below the smallest size standard (10 kDa) at the end of the gel. This result is consistent with that observed by sequencing by nanoLC-MS/MS in which the mass of the largest peptide was 4.5 kda,

A total of 24 *H. capsulatum* proteins were identified in the BMDM samples, while BMDCs samples contained 45 *H. capsulatum* proteins (**Figure 3C**). Twenty-two of the 24 proteins presented by BMDM were also presented by BMDCs. Additionally, 4 proteins were identified and presented by both cell types in the Co-IP and visualized in 2-D gel: Hsp60-like protein (HCBG_08832), malate dehydrogenase (HCEG_05915), fructose-bisphosphate aldolase (HCBG_06745), and ATP synthase subunit alpha (HCBG_01891). Enolase (HCEG_02034) was presented by DCs and also identified by 2-D PAGE (**Figure 3C**). Regarding peptides, 42 distinct sequences of *H. capsulatum* were presented by BMDM and 74 by BMDC. Forty *H. capsulatum* peptide sequences were presented by both APCs (**Figure 3D**). All proteins corresponding to the peptide sequences presented exclusively by BMDM or BMDCs and peptides commonly presented by both cells are shown in **Table 2**.

**Table 2:** MHC-II-binding *H. capsulatum* peptides from BMDC (DCs) and BMDM (Mφ) bone marrow-derived from C57BL/6 mice.

Protein name	Peptide sequence	APCs^a^	Accession number^b^
**Hsp60-like protein**	TALVDASGVASLLGTTEVAIVEAPEEK TNEVAGDGTTTATVLAR	Mφ and DC	HCBG_08832
**Fructose-bisphosphate aldolase**	VNLDTDMQYAYLSGVR VQEGLDDFNTSGQL DYLLTAVGNPEGDDKPNKK GYAIPAINVTSSSTVVAALEAAR KTGVIVGDDVLR SPLILQVSQGGAAFFAGK TGVIVGDDVLR	Mφ and DC	HCBG_06745
**Malate dehydrogenase**	ANILVISNPVNSTVPIVAEVFK DVIEPTFVDSPLYK GGPGVAADLSHINTNSTVTGYDPTPSGLR LFGVTTLDVVR VTVLGAAGGIGQPLSLLMK	Mφ and DC	HCEG_05915
**Nascent polypeptide-associated complex subunit alpha**	GILFVINQPDVYR SPSSNTWIIFGEAK	Mφ and DC	HCBG_02857
**ATP synthase subunit alpha**	TGEIVDVPVGPELLGR	Mφ and DC	HCBG_01891
**Glyceraldehyde-3-phosphate dehydrogenase**	VLSNASCTTNCLAPLAK	Mφ and DC	HCEG_09258
**Pfs domain-containing protein**	QLSGSQTVTPFSLFSR	Mφ and DC	HCBG_07657
**Ribosomal protein L37**	HTCSSCGYPSAK	Mφ and DC	HCEG_00999
**Ribosomal protein L31e**	LYSFVQAVNVK LYSFVQAVNVKEPK	Mφ and DC	HCAG_05192
**Ribosomal protein L7a**	KVATAPFPQGK LKVPPAIAQFQNTLDR	Mφ and DC	HCAG_05221
**Ribosomal protein L14**	VLVDGPAGQENK HVLALSHATLTPFTIPK	Mφ and DC	HCBG_02856
**40S ribosomal protein S7**	FEFPQSSATEF QNPSELETSLAGALSDLETNTPDLK	Mφ and DC	HCEG_02939
**40S ribosomal protein S5**	SIAECLAEELINAAK	Mφ and DC	HCEG_03546
**40S ribosomal protein S7**	QNPSELETSLAGALSDLETNTPDLK	Mφ and DC	HCDG_03631
**60S ribosomal protein L36**	KVDEMQGVIAEAK KVDEMQGVIAEAKR RKVDEMQGVIAEAK VDEMQGVIAEAK	Mφ and DC	HCBG_00063
**60S ribosomal protein**	QLDELKTELGQLR	Mφ and DC	HCEG_02183
**60S ribosomal protein**	AVVGASLDVIK	Mφ and DC	HCEG_08256
**60S ribosomal protein**	IIVHPLNTESAMK LTPDVDALDIAATK	Mφ and DC	HCEG_04281
**60S ribosomal protein L31**	LYSFVQAVNVK	Mφ and DC	HCDG_02034
**Uncharacterized protein**	ENQSAHTISQR	Mφ and DC	HCDG_06427
**Uncharacterized protein**	DPLQLLDELIASGR	Mφ and DC	HCBG_06739
**Uncharacterized protein**	LSGAIMTYDRR	Mφ and DC	HCAG_07106
**ATP-dependent molecular chaperone HSC82**	GVVDSEDLPLNLSR	Mφ	HCAG_04686
**Lipase/serine esterase**	NLEMEQTEIATESQR	Mφ	HCDG_00413
**Enolase**	TADCQIVGDDITVTNPLR	DC	HCEG_02034
**Vacuolar protein sorting-associated protein 35**	MASPPNVPEEQSR	DC	HCEG_00302
**Alcohol dehydrogenase**	LDEVAPILCAGVTVYK TVELSELGNVFSLMK ESGAKPGQAVAIVGAGGGLGSLAQQYAK	DC	HCEG_08061
**Serine hydroxymethyltransferase**	YYGGNQFIDQAER TYQETVLENAK	DC	HCAG_07418
**Isochorismatase domain-containing protein**	VYVVADGVSSVNPEER	DC	HCAG_07305
**Mitochondrial F1F0 ATP synthase subunit F Atp17**	VASPTGIGAAQDAAR	DC	HCEG_06769
**3-hydroxybutyryl CoA dehydrogenase**	AGVPVTLIDNSQASIDK	DC	HCEG_04858
**Sm domain-containing protein**	GTQVVSCSVDGPPPADPAAR	DC	HCBG_00112
**H15 domain-containing protein**	INITSQSAFDTQFNR KAPVAPAVVDAPK	DC	HCEG_07158
**Ribosomal protein S23**	VTAFVPNDGCLNFVDENDEVLLAGFGR	DC	HCEG_08043
**Ribosomal protein L37a**	VVAGGAWTVSTPAAATIR	DC	HCEG_03019
**Ribosomal protein L14**	AQIDITTSSWK HVLALSHATLTPFTIPK	DC	HCDG_01939
**40S ribosomal protein S15**	GIDLNQLLDLSSEQLR	DC	HCBG_01774
**60S ribosomal protein L2**	GMVGIVAGGGR NATLTVGNILPLGSVPEGTVMTNVEEK TSGNYVTVIGHNPEEGK	DC	HCDG_08240
**60S ribosomal protein L13**	VNASTESLTLNVAR	DC	HCAG_07708
**60S ribosomal protein L12**	QAQVSVVPSASSLVIK SIPLDEIIEIAR	DC	HCDG_03181
**60S ribosomal protein L27**	VVIIQPYDAGSK	DC	HCEG_00238
**60S ribosomal protein**	AGEQTSAESWGTGR AGQAAFGNQCR DFLLPSNLISNADITR FATASALAASSVPALLYAR	DC	HCEG_06198
**60S ribosomal protein**	AGGETLTLDQLALR	DC	HCEG_01279
**60s ribosomal protein**	TLINNLIIGVTR	DC	HCEG_08000
**Mitochondrial thiamine pyrophosphate carrier 1**	LLIQNQDEMLK YFPTQALNFAFR	DC	HCDG_00690
**Uncharacterized protein**	LKPQQAPVVNPQDIR	DC	HCEG_00555
**Uncharacterized protein**	IIVSGLPSDVGEANIK	DC	HCEG_05998

a APCs, Antigen presenting cells.

b Access number to Uniprot database.

The proteins obtained by Co-IP are shown and classified in **Table S2**, with the exception of the common proteins described in **Table 1** identified by 2-D PAGE.

### 3.4 Identical and Non-Identical Antigens

The proteins were analyzed comparatively for identity to human proteins and other important pathogenic fungal species to identify exclusive or conserved molecules. BLAST-p algorithm was used to compare the proteins of *H. capsulatum* against *Homo sapiens* and other pathogens with genomes annotated in the NCBI database and proteins deposited in the Uniprot database, including *P. brasiliensis*, *B. dermatitidis, C. immitis*, *S. schenckii*, *A. fumigatus*, *C. neoformans*, *C. albicans* and *C. auris*. The proteins chosen for this analysis are considered stable and hydrophilic, or constituent of the plasma membrane. *H. capsulatum* proteins identified by both techniques, 2-D PAGE and Co-IP, were included.

**Figure 4:**
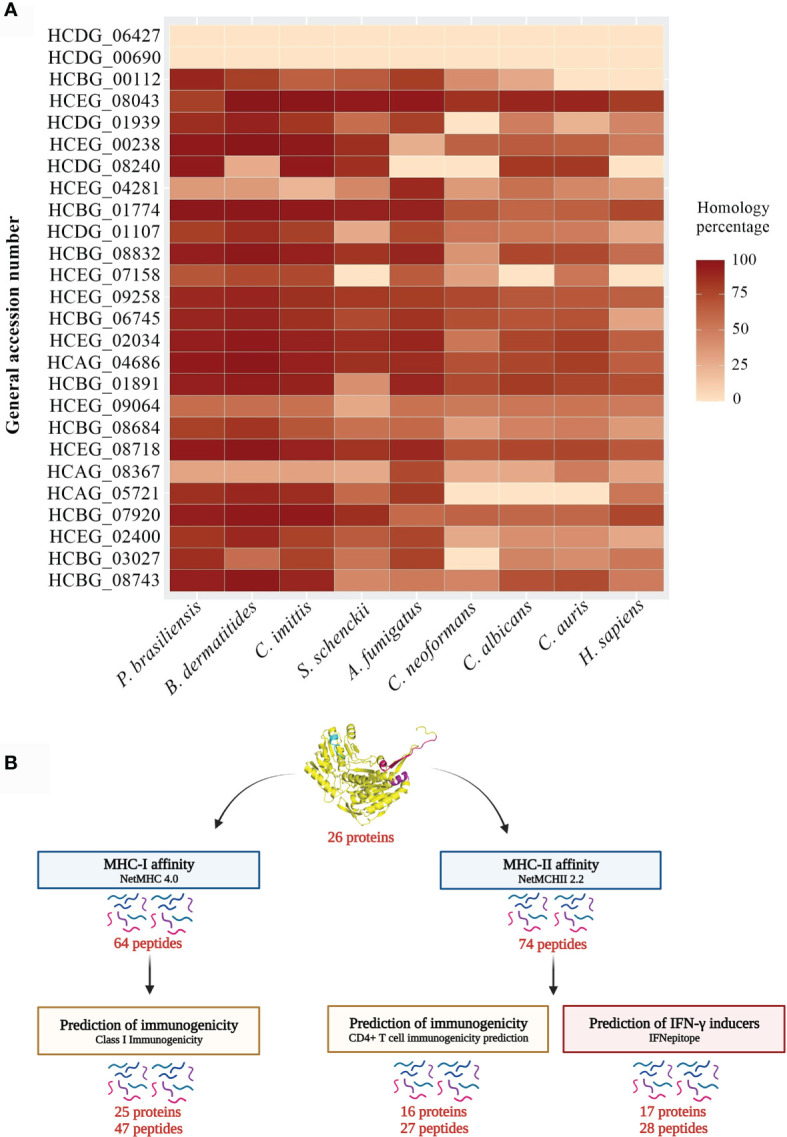
Heat map and bioinformatics analysis flowchart. **(A)** Heat map of the similarity percentage of 26 *H. capsulatum* antigenic proteins against *P. brasiliensis*, *B. dermatitidis*, *C. immitis*, *S. schenckii*, *A. fumigatus*, *C. neoformans*, *C. albicans*, *C. auris* and *H. sapiens* and bioinformatics analysis flowchart. The analysis was performed by BLAST-p algorithm and ClustalX 2.1 software and the map plotted using software R package ggplot. **(B)** (I) Prediction of ligands to MHC-I was performed using NetMHC 4.0 server from the sequences of the 26 antigenic proteins previously described. (II) The NetMHCII 2.2 Server was used to predict MHC-II ligands, including the peptide sequences obtained by immunoprecipitation and that had low or no identity to human protein. (III) The Class I Immunogenicity software was used to predict the immunogenicity of the class I peptide MHC complex. (IV) The prediction of immunogenicity for MHC-II was performed by CD4^+^ T cell immunogenicity prediction server. (V) The IFNepitope server was used to predict IFN-γ induction by MHC-II binding peptides. HCDG_06427: Uncharacterized protein, HCDG_00690: Mitochondrial thiamine pyrophosphate carrier 1, HCBG_00112: Sm domain-containing protein, HCEG_08043: Ribosomal protein S23, HCDG_01939: Ribosomal protein L14, HCEG_00238: 60S ribosomal protein L27, HCDG_08240: 60S ribosomal protein L2, HCEG_04281: 60S ribosomal protein, HCBG_01774: 40S ribosomal protein S15, HCDG_01107: Peroxidase, HCBG_08832: HSP-60 like protein, HCEG_07158: H15 domain-containing protein, HCEG_09258: Glyceraldehyde-3-phosphate dehydrogenase, HCBG_06745: Fructose-bisphosphate aldolase, HCEG_02034: Enolase, HCAG_04686: ATP-dependent molecular chaperone HSC82, HCBG_01891: ATP synthase subunit alpha, HCEG_09064: Citrate synthase, HCBG_08684: Elongation factor 1-gamma, HCEG_08718: 6-Phosphogluconate dehydrogenase, HCAG_08367: Aldehyde dehydrogenase, HCAG_05721: Homogentisate 1,2-dioxygenase, HCBG_07920: Hsp70-like protein, HCEG_02400: Aha1_N domain-containing protein, HCBG_03027: 4-Hydroxyphenylpyruvate dioxygenase, HCBG_08743: Heat shock protein SSC1.

In total, 26 proteins were analyzed and the results were plotted on the heat map shown in **Figure 4A**. Notably, the uncharacterized protein (HCDG_06427) and mitochondrial thiamine pyrophosphate carrier 1 (HCDG_00690) do not have identity with proteins of the other evaluated pathogenic fungi. However, HCDG_06427 shows 56.76% similarity with the UPF0495 protein YPR010C-A from *Saccharomyces cerevisiae*, a protein located in the plasma membrane. HCDG_00690 is a transmembrane transporter that presents identity of 80.91% with the carrier protein/Adenine nucleotide translocator of *Neurospora crassa*.

In general, all analyzed proteins showed some degree of similarity with antigens from other fungi and/or humans. These data reinforce the relevance of epitope selection for these targets.

### 3.5 Prediction of Peptides With Affinity to MHC-I and II

MHC slits are closed at both ends by conserved tyrosine residues that restrict the size of peptides that can bind to the slit. The MHC class I pathway results in an antigen with short peptide sequences, from 8 to 10 amino acids, while the MHC-II pathway can results in longer peptide sequences, generally from 13 to 25 amino acids (32). We used bioinformatics tools to predict the affinity of *H. capsulatum* peptide binding to MHC-I and II slit, epitopes of T cells and, to determine the potential of these epitopes to generate an immune response (see **Figure 4B** for more details).

The identified sequences of MHC-I and -II epitopes shown in **Tables S3, S4**, respectively, satisfy the following selection criteria: (I) affinity values indicating strong binders (SB) and/or weak binders (WB), for MCH-I and SB and intermediate binders (IB) for MHC-II alleles from humans and mice, (II) promiscuity, and (III) peptides with low or no affinity for human protein, in which the similarity percentage was identified in the third column of **Table S3**.

### 3.6 Prediction of Immunogenicity

The epitopes restricted to MHC-I and II described in **Tables S2, S3** were predicted according to the probability of generating an immune response using The Class I Immunogenicity and CD4^+^ T cell immunogenicity prediction server (**Figure 4B**). The results can be seen in **Table S5**.

The activation of naive helper T lymphocytes after presentation of peptides by APCs is mediated by the production of IFN-γ, which is essential to generate a protective response of type Th1 (33). The IFNepitope server was used to predict IFN-γ induction by MHC-II binding peptides and the results are described in **Table S6**. These results demonstrate that some of the selected epitopes are predicted to induce cell-mediated immune response.

### 3.7 Peptides Similar to Other Fungi

**Figure 5:**
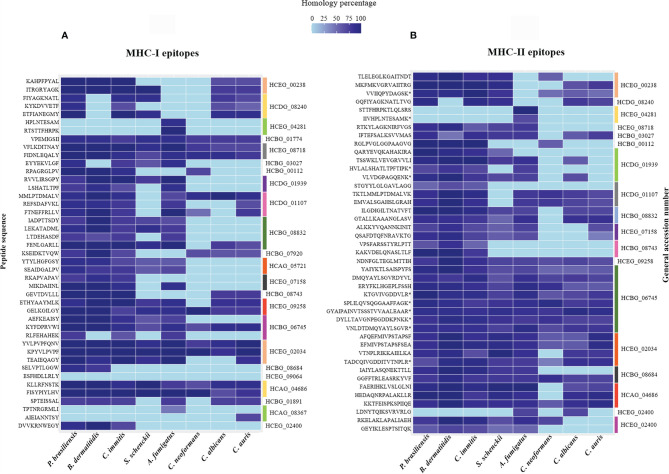
Heat map of the percentage of similarity of epitopes derived from *H. capsulatum* antigenic proteins against *P. brasiliensis*, *B. dermatitidis*, *C. immitis*, *S. schenckii*, *A*. *fumigatus*, *C. neoformans*, *C*. *albicans* and *C. auris*. **(A)** Similarity percentage of 43 MHC I-binding epitopes derived from 23 antigenic proteins. **(B)** Similarity percentage of 43 MHC II-binding epitopes derived from 18 antigenic proteins. The analysis were performed by BLAST-p algorithm and the map was plotted using software R package ggplot. HCEG_00238: 60S ribosomal protein L27, HCDG_08240: 60S ribosomal protein L2, HCEG_04281: 60S ribosomal protein, HCBG_01774: 40S ribosomal protein S15, HCEG_08718: 6-Phosphogluconate dehydrogenase, HCBG_03027: 4-Hydroxyphenylpyruvate dioxygenase, HCBG_00112: Sm domain-containing protein, HCDG_01939: Ribosomal protein L14, HCDG_01107: Peroxidase, HCBG_08832: HSP-60 like protein, HCBG_07920: Hsp70-like protein, HCAG_05721: Homogentisate 1,2-dioxygenase, HCEG_07158: H15 domain-containing protein, HCBG_08743: Heat shock protein SSC1, HCEG_09258: Glyceraldehyde-3-phosphate dehydrogenase, HCBG_06745: Fructose-bisphosphate aldolase, HCEG_02034: Enolase, HCBG_08684: Elongation factor 1-gamma, HCEG_09064: Citrate synthase, HCAG_04686: ATP-dependent molecular chaperone HSC82, HCBG_01891: ATP synthase subunit alpha, HCAG_08367: Aldehyde dehydrogenase, HCEG_02400: Aha1_N domain-containing protein. (*) Sequences of peptides obtained through the method of immunoprecipitation that were naturally presented by murine BMDMs and BMDCs.

To identify *H. capsulatum* peptides similar to those produced by other pathogenic fungi, the immunogenic MHC-I and II ligand epitopes were analyzed comparatively with proteins from other fungal species using the BLAST-p algorithm. As shown in **Figure 5**, sequences of epitopes with 100% similar to other species of fungi were observed.

### 3.8 Prediction and Validation of 3-D Structure and Location of Epitopes Restricted to MHC-I and II

Proteins have two types of epitopes, cryptic and dominant. Dominant epitopes are exposed on the surface of the protein and are more likely to generate an immune response, while cryptic epitopes are more hidden in the protein structure and can be recognized but result in weaker immune responses (34). Therefore, it is essential that we understand the location of these peptides in the entire protein. Of note, bioinformatics analyses were performed to discover linear epitopes. Thus, the 3-D structure is independent with regard to the later activity of these peptides. The 3-D structures of the identified immunogenic *H. capsulatum* proteins were built using I-TASSER server and the models refined by GalaxyRefine algorithms. The location of promising peptide sequences was highlighted using PyMOL software (**Figures S1A–G**). **Figures S1H–K** shows protein structures with epitopes exclusive to *H. capsulatum*. We have observed that most epitopes identified were exposed on the surface of the protein and, therefore, we may be dealing with the dominant epitopes of these proteins, responsible for inducing a more robust immune response. The validation of the structures was performed using PROCHECK server and the generated ramachandran plots are displayed in **Figure S2**.

### 3.9 Peptides

**Figure 6:**
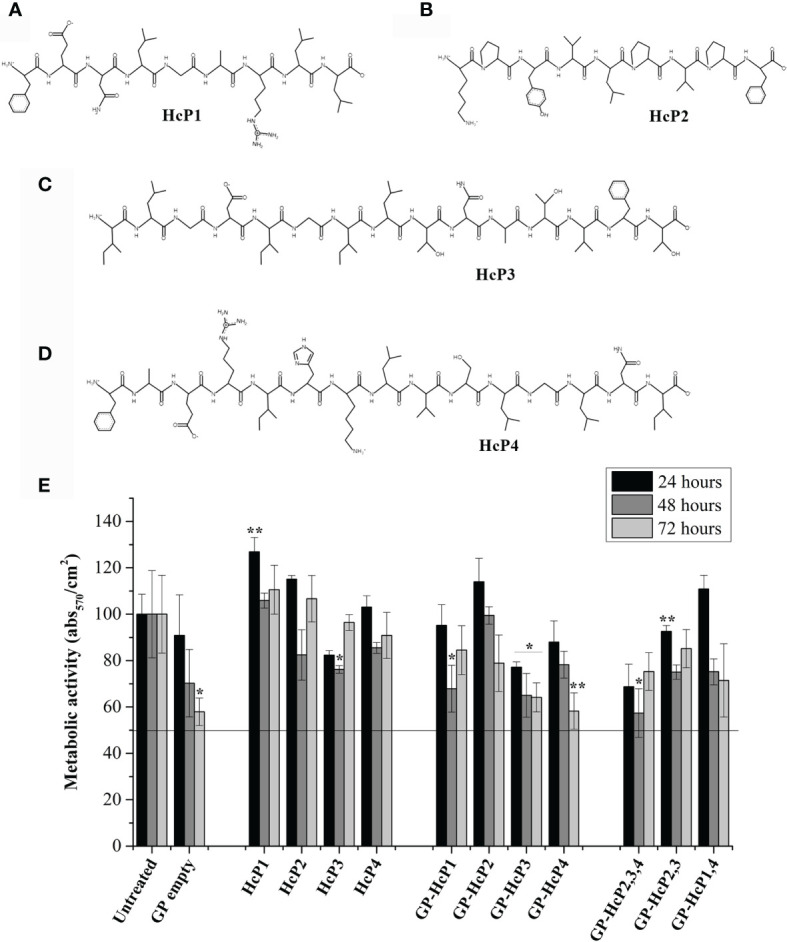
Chemical structure of the synthesized peptides and their effects on the viability of the Jaws II cells. The chemical structures of **(A)** HcP1 (HSP60), **(B)** HcP2 (enolase), **(C)** HcP3 (HSP60), and **(D)** HcP4 (HSC82) were obtained by PepDraw server. **(E)** Effects of HcP 1-4 tested from 10 µg/200µL, GP-HcP 1-4 and GP-HcP 2,3,4; 2,3 or 1,4 tested at 5 µg/200µL for 24 and 48 hours. The percent cell viability (%CV) was calculated from the absorbance values obtained by the microplate reader at 570 nm, according to the following equation: %CV = (Treated sample/Blank) x P100, where Blank and P100 represent, respectively, the negative control containing only culture medium and positive control containing the untreated cells, considered to be 100% cell viability. Difference from positive control by the Tukey’s test, *p < 0.05 or **p < 0.01.

Four peptides (**Table 3**) were chosen and synthesized for meeting the following criteria: (I) no similarity to human proteins, (II) similarity with at least 2 other species of pathogenic fungi (III) capable of binding to the MHC-I or II slit of humans or mice, and (IV) with potential to induce immune response through recognition by CD4^+^ T or CD8^+^ T lymphocytes. The alignment of the protein sequences of *H. capsulatum*, other fungi and *Homo sapiens* are shown in **Figure S3** where the location of each peptide is highlighted. The chemical structures of these peptides were constructed using PepDraw and are shown in **Figures 6A–D**. The peptides were diluted and incorporated into glucan particles (GP) for cytotoxicity tests on Jaws II cells (**Figure 6E**). A MTT reduction assay was used to assess toxicity. In general, compounds are considered cytotoxic when the concentration evaluated inhibits 50% of cell growth (IC_50%_). None of the evaluated concentrations of peptide or peptide incorporated into GP were considered cytotoxic.

**Table 3:** Peptides chosen for synthesis and biological testing and their respective characteristics.

Characteristic of the peptide	HcP1^a^	HcP2	HcP3	HcP4
**Sequence**	FENLGARLL	KPYVLPVPF	ILGDIGILTNATVFT	FAERIHKLVSLGLNI
**Protein of origin**	HSP60	Enolase	HSP60	HSC82
**Net Charge at pH 7**	Neutral	Neutral	Acid	Basic
**Molecular weight**	1032.2 g/mol	1059.31 g/mol	1547.8 g/mol	1710.04 g/mol
**pI**	6.34	9.3	3.75	9.69
**100% similar**	*P. brasiliensis* *C. immitis* *B. dermatitidis* *A. fumigatus*	*P. brasiliensis* *C. immitis* *B. dermatitidis* *S. schenckii* *A. fumigatus* *C. neoformans* *C. auris*	*P. brasiliensis* *B. dermatitidis*	*B. dermatitidis* *S. schenckii* *A. fumigatus*
**% similarity with others**	*S. schenckii* (88.89%)	*C. albicans* (85.71%)	*C. immitis* (93.33%)	*P. brasiliensis* *C. immitis* (93.33%)
**Subcellular location described in fungi**	Mitochondrial and cell wall (35)	Cytosol and cell wall (36).	Mitochondrial and cell wall (35)	Cytoplasmatic (37)

a **HcP1,** H. capsulatum peptide 1.

### 3.10 Peptides From HSP60, Enolase, and HSC82 Change the Shape of GPs and Induce Lymphocyte Proliferation With Secretion of IFN-γ and IL-17

**Figure 7:**
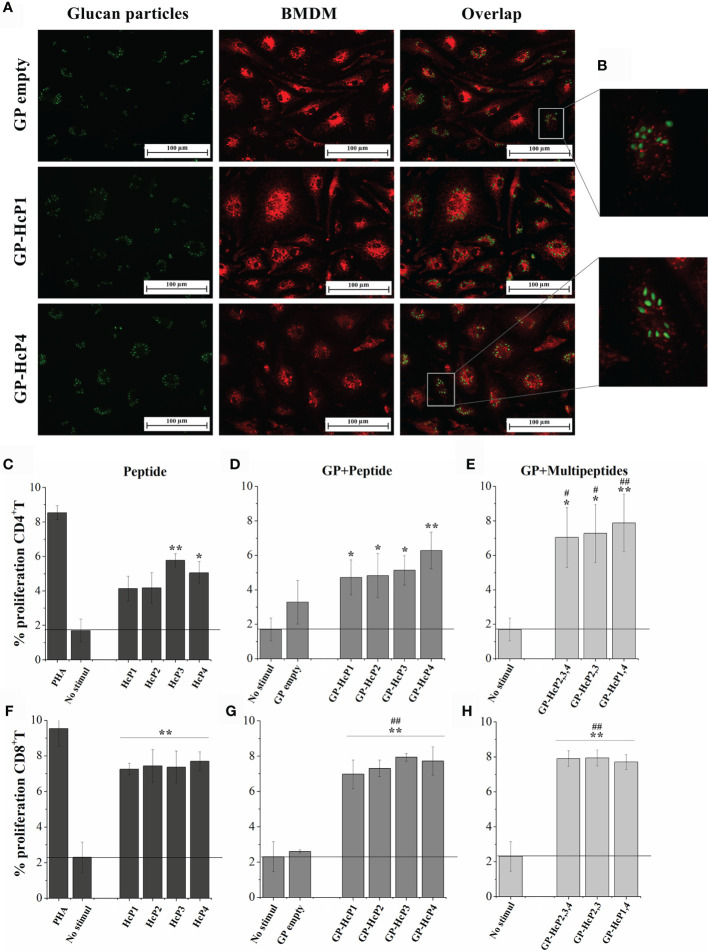
Phagocytosis and proliferation of CD4^+^ and CD8^+^ T lymphocytes after exposure to the peptides and formulations of GPs. **(A)** Fluorescence microscopy of GPs empty and GP complexed with HcP1 and HcP4 (stained in green) ingested by BMDM (stained in red). Overlap: Both images, with GPs and BMDM, represent the same quadrant of the culture plate well according to the phagocytosis time of 3 hours. **(B)** Quadrant enlargement showing the shape of empty GPs and GP-HcP4. C-H. Splenocytes were collected from mice infected with *H*. *capsulatum* and marked with CFSE for re-stimulated with: **(C, F)** 10 µg/200µL HcP 1-4, **(D, G)** 5 µg/200µL GP-HcP 1-4 and **(E, H)** 5 µg/200µL GP-HcP 2,3,4; 2,3 or 1,4 for 96 hours. Control of cells stimulated with mitogen (PHA) and without stimuli were used for validation of the proliferative capacitive. Data shown are representative of four independent experiments. Differ from positive control by the Tukey’s test, *p < 0.05 or **p < 0.01. Differ from GP empty by the Tukey’s test, ^#^p < 0.05 or ^##^p < 0.01.

**Figure 8:**
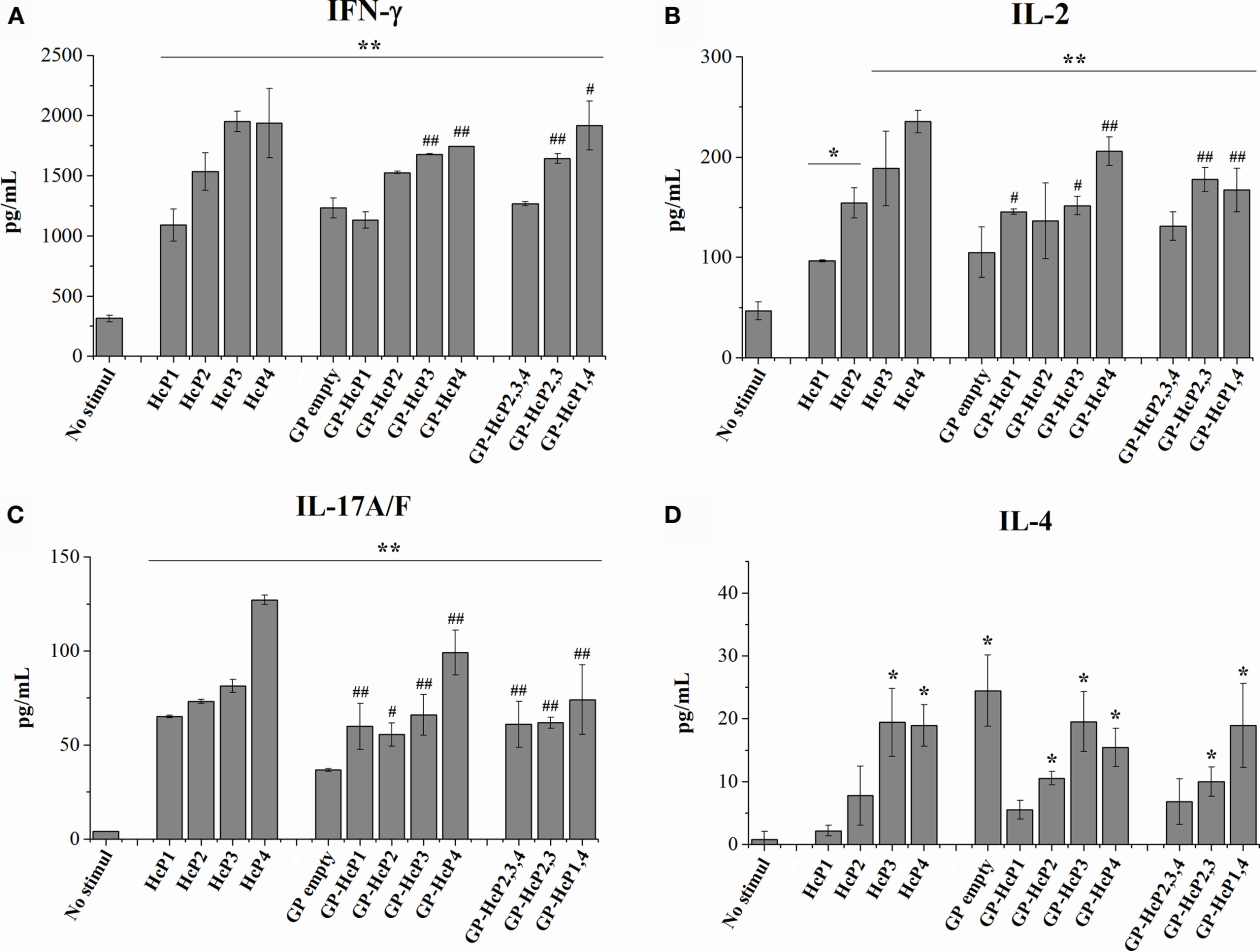
Cytokines levels of **(A)** IFN-γ, **(B)** IL-2, **(C)** IL-17 and **(D)** IL-4 after 72 hours of lymphoproliferation. HcP 1-4 was tested to 10 µg/200µL, GP-HcP 1-4 and GP-HcP 2,3,4; 2,3 or 1,4 was tested to 5 µg/200µL. Data shown are representative of two independent experiments. Difference from positive control by the Tukey’s test, *p < 0.05 or **p < 0.01. Difference from GP empty by the Tukey’s test, ^#^p < 0.05 or ^##^p < 0.01.

First, empty GPs complexed with peptides were validated for their potential to be phagocyted by BMDMs. Phagocytosis of empty GPs and GPs complexed with FITC-labeled HcP1 (9-mer) and HcP4 (15-mer) peptides are shown in **Figure 7A**. All GPs were taken up efficiently by BMDMs. The fluorescence staining revealed a change in the shape of the GPs, in which it is noted that the empty GPs have a predominantly rounded shape, while most of the GPs complexed with the peptides acquired an oval shape **(** **Figure 7B**). **Figures 7C–E** shows re-stimulation of CD4^+^ T from the spleen and lymph nodes of mice. Pure HcP3 and HcP4 peptides promoted the significant increases in lymphoproliferation of CD4^+^ cells. All peptides incorporated into GPs significantly stimulated lymphoproliferation of CD4^+^ cells and GPs loaded with one more peptide (multi peptides) produced even more robust responses. All pure or complexed GP- complexed peptides significantly stimulated CD8^+^ T cells (**Figures 7F–H**). The cytometry gate strategy is shown in **Figure S4**. The pure and GP-complexed peptides stimulated the secretion of high levels of IFN-γ, IL-17 and IL-2 (**Figure 8**). HcP3 and HcP4 induced expression of IL-4. However, the amounts of this cytokine were relatively lower, indicating a predominantly Th1 and Th17 response. Compared to GP alone, HcP1 and HcP2 expressed low amounts of IL-4, along with GP-HcP1, GP-HcP2, & GP2,3,4 and GP2,3. Suggesting that the presence of peptides HcP1 and HcP2 may be related to the low expression of IL-4.

## 4 Discussion

Techniques based on proteomics and bioinformatics have enabled the scientific community to map metabolic pathways and discover targets for the development of new drugs, therapies, and biological markers for diagnosis (38). In this study, we used two distinct proteomic techniques. The first was based on immunoproteomics, in which the combination of 2-D gel and immunoblot allowed the identification of antigenic proteins of *H. capsulatum*. The second was based on immunopeptidomics, in which the MHC-II Co-IP of DCs and Mφ from mice after phagocytosis allowed the isolation of *H. capsulatum* peptides with the potential to be immunogenic.

Peptide vaccines are based on short, relevant protein fragments that make the formulation safer and more stable when applied in a suitable adjuvant, and are easier to produce compared to complete recombinant proteins (39). In our study, we describe that the combination of 2-D electrophoresis and immunoblotting is a valuable tool for assessing the profile of immune responses against *H. capsulatum* proteins and identifying antigenic targets, as previously demonstrated for *A. fumigatus* (38) and *Sporothrix* spp (40). Through these techniques, we identified 17 antigenic proteins of *H. capsulatum*. We then used bioinformatics to predict peptide vaccine candidates.

Peptide vaccines for human use must contain epitopes restricted to HLA-I and HLA-II to ensure the induction of a balanced and protective immune response against fungal infections involving CD4^+^ and CD8^+^ T lymphocytes. The immunoprotective role of CD4^+^ T cells is underscored by their absence in individuals with AIDS, resulting in a high risk for developing disseminated fungal infections (41). In addition, both CD4^+^ and CD8^+^ T cells are necessary to prevent the reactivation of certain fungal infections (42). Thus, we selected promiscuous epitopes, that is, peptides predicted to bind to several histocompatibility alleles, in this case to MHC-I and -II alleles of humans and mice, to provide improved coverage of the world’s population. We also included animals, which will be utilized to validate the efficacy of the peptide candidates.

Peptide antigens linked to MHC molecules are targets for T lymphocytes (21). Proper identification of peptide antigens is crucial for developing vaccines and immunotherapies that produce efficient immune responses and long-term immune memory (21, 43). We developed a protocol for the isolation and identification of *H. capsulatum* peptides naturally presented on the surface of BMDM and BMDC using nanoLC-MS/MS. This technique has a limitation in which, for small samples, the most abundant and most easily detectable (ionizable) peptides will be identified. Therefore, we chose to use differentiated mouse bone marrow cells since it is possible to extract large amounts of cells (24). The first step was to evaluate the typical rates of phagocytosis of *H. capsulatum* by the host effectors cells, and approximately 75% of the yeasts were ingested by 4 hours of co-culture. These results are consistent with a previous report showing that *H. capsulatum* yeasts are degraded after 2 hours of phagocytosis by human DCs (44). In our study, the extraction of the MHCII-peptide complex was performed after 4 hours of phagocytosis, which was also the threshold for MHC-II expression.

A total of 76 peptides derived from 47 different proteins were obtained by Co-IP. Co-IP is a valuable tool for identifying possible antigenic targets for the development of vaccines and immunotherapies (21). However, not everything presented by an APC will lead to efficient proliferation of T cells, such as irrelevant MHC-peptide complexes (45). In addition, there are several regions in proteins containing dominant epitopes, which are capable of initiating potent T cell responses (34). Therefore, the application of bioinformatics analysis combined with proteomics tools is necessary for effectively screening and selecting the most promising epitope candidates.

Notably, most of the proteins identified with immunogenic potential are historically associated only with intracellular activity. However, in the last decade, studies on the composition of the fungal cell wall have shown that this structure is not only composed of polysaccharides, glycoproteins and pigments, but that atypical molecules, normally located in the intracellular space, are also present (46). Therefore, the fungal cell wall has a very high molecular diversity. Important enzymes for fungal metabolism emerged in our analysis and have been described as potentially immunogenic and are found in the cell walls of different species, which include glyceraldehyde-3-phosphate dehydrogenase (47), HSP70 (48), HSP60 (35) and fructose-bisphosphate aldolase (49). Therefore, the appearance of these and other proteins in our analyses confirms the robustness of proteomics approaches for the screening and discovery of new fungal antigens.

In this study, bioinformatics analyses resulted in the identification of promiscuous epitopes with immunogenic potential and/or for inducing IFN-γ that lack similarity with human proteins. The most promising peptide sequences obtained were compared regarding the percentage of similarity with the same proteins expressed by other fungi. Four peptides that best fit these criteria were synthesized. Notably, the epitopes selected for synthesis have 100% identity in up to 7 other species of fungi: *P. brasiliensis*, *B. dermatitidis*, *S. schenckii*, *C. immitis*, *C. neoformans*, *C. auris* and *A. fumigatus*. Thus, the use of these epitopes has the potential to generate cross-protection between for all these species, in addition to *H. capsulatum*. Two protein antigens selected in this study, HSP60 and enolase, have already been reported as potential vaccine targets using recombinant proteins (6, 50). Nonetheless, the specific peptides presented by APCs were not identified in these previous studies.

HSP60 is an immunodominant antigen expressed on the surface of *H. capsulatum*, and is an important protein related to pathogenesis, since it mediates entry into macrophages through its recognition by the CR3 receptor (CD11b/CD18) (35, 51). In our study, HSP60 was recognized by antibodies from infected mice (2-D immunoproteomic data) and it was also presented *via* MHC-II by BMDM and BMDC (immunopeptidome data). Ours results confirm the efficacy of applying both proteomics strategies to identify and screen the antigens of *H. capsulatum.* Monoclonal antibodies against HSP60 are capable of inducing a protective immune response against *H. capsulatum* infection (46). Vaccination strategies using recombinant protein (HSP60) from *Histoplasma* or *P. brasiliensis* protect mice from infection by reducing fungal load and increasing IFN-γ production (52–54). These results reinforce the potential of this antigen as a vaccine target. In this study, re-stimulation of splenocytes with HcP1 and HcP3 (HSP60 epitopes) in free or GP-carried form promoted the lymphoproliferation of CD4^+^ and CD8^+^ T lymphocytes, with the GP-carried peptides (microparticles) demonstrating a more robust proliferative response. This improvement in the immune response can be explained by the targeted delivery promoted by the β-glucan carrier *via* Dectin-1. Furthermore, the microparticle may protect the peptide from degradation by extra- and intracellular enzymes (1, 55).

Enolase (Eno) is a metalloenzyme responsible for the formation of phosphoenolpyruvate (PEP), a product used in the synthesis of ATP by eukaryotic and prokaryotic cells. Immunization with rEno formulated in Montanide Pet-GelA (PGA) protected mice from *S. brasiliensis*, and the immunization induced a Th1-type response and high levels of IFN-γ and IL-2 (50). In our work, splenocyte stimulation with Eno’s HcP2 epitope resulted in proliferation of CD4^+^ and CD8^+^ T lymphocytes. Moreover, this stimulation induced high levels of IFN-γ, IL-17 and IL-2, with IL-2 produced only when the antigen was carried by GPs. Interestingly, Eno is also displayed on the cell surface of non-fungal pathogens, such as *Plasmodium* spp, *Ascaris suum* and *Streptococcus sobrinus* (56–58). Thus, Eno may be an excellent protective antigenic target shared between prokaryotic and eukaryotic pathogens.

*S. cerevisiae* has two proteoforms of HPS90 that differ in structure and function. The proteoform HSP82 is induced by stress and the other proteoform, HSC82, is expressed constitutively (37). In *H. capsulatum*, HSP82 has been identified during the conversion of the mycelium to yeast form and it is actively transcribed at 37°C (59). The reduction of the expression of the HSP82 gene results in decreased virulence. Therefore, *H. capsulatum* HSP82 is closely related to survival during thermal shock and the virulence capacity (59). However, little is known about *H. capsulatum* HSC82 and its role in virulence. Nevertheless, this protein has a promising immunogenic potential that leads to CD4^+^ T cell lymphoproliferation both as a free peptide and/or complexed to GPs. As observed with HcP2, the proliferation of CD8^+^ T cells was promoted when the peptide was incorporated into GPs.

In conclusion, we identified *H. capsulatum* peptide epitopes, derived from HSP60, enolase, and HSC82 proteins restricted to MHC-I and MHC–II with high promiscuity that are also expressed by several additional important fungal pathogens. These epitopes were tested separately and incorporated into GPs, and the peptides efficiently induced the proliferation of CD4^+^ and CD8^+^ T lymphocytes, and stimulated the production of IFN-γ and IL-17. The results presented in this study constitute an advance in the identification of T cell epitopes that can be explored individually or together for the development of a multi-epitope peptide vaccine to combat *H. capsulatum* and other fungi. Moreover, our positive findings support the next steps of our planned study: determining which peptides will be part of the vaccine composition, selecting adjuvants, and determining the immunization route that provides the most protective activation of the immune system in prophylactic and therapeutic animal histoplasmosis models followed by other systemic mycoses models.

## Data Availability Statement

The datasets presented in this study can be found in online repositories. The names of the repository/repositories and accession number(s) can be found in the article/**Supplementary Material**.

## Ethics Statement

The animal study was reviewed and approved by Ethic Committee on Animal Use (number 1169061218) of the Biomedical Sciences Institute (CEUA-ICB/USP).

## Author Contributions

BK conducted the experiments, collected and analyzed the data and wrote the manuscript. CB and IG collected the data. SR, CA, and GP analyzed the data. SA, GP, LL-B, JN, and CT reviewed the manuscript. All authors contributed to the article and approved the submitted version.

## Funding

This research was funded by São Paulo Research Foundation (FAPESP) grants 2016/08730-6, 2018/26402-1, 2014/06863-3, 2018/18257-1, 2018/15549-1 and 2017/25780-0. CNPq (grant 420480/2018-8) and CAPES-Education Ministry, Brazil. CT and GP are research fellows of the CNPq.

## Conflict of Interest

The authors declare that the research was conducted in the absence of any commercial or financial relationships that could be construed as a potential conflict of interest.

## Publisher’s Note

All claims expressed in this article are solely those of the authors and do not necessarily represent those of their affiliated organizations, or those of the publisher, the editors and the reviewers. Any product that may be evaluated in this article, or claim that may be made by its manufacturer, is not guaranteed or endorsed by the publisher.
