# Supplementary material for: Immunoproteomic and Immunopeptidomic Analyses of Histoplasma capsulatum Reveal Promiscuous and Conserved Epitopes Among Fungi With Vaccine Potential
Source: Front Immunol. 2021 Nov 22;12:764501. doi: 10.3389/fimmu.2021.764501 (PMC8645968; doi:10.3389/fimmu.2021.764501)
Supplement: Supplementary file 1 [file DataSheet_1.docx]

***Supplementary Material***

The raw data were deposited in the PRIDE mass spectrometry-based protein identification database (<https://www.ebi.ac.uk/pride/>). Dataset identifier: PXD024431.

1. **Supplementary Figures**


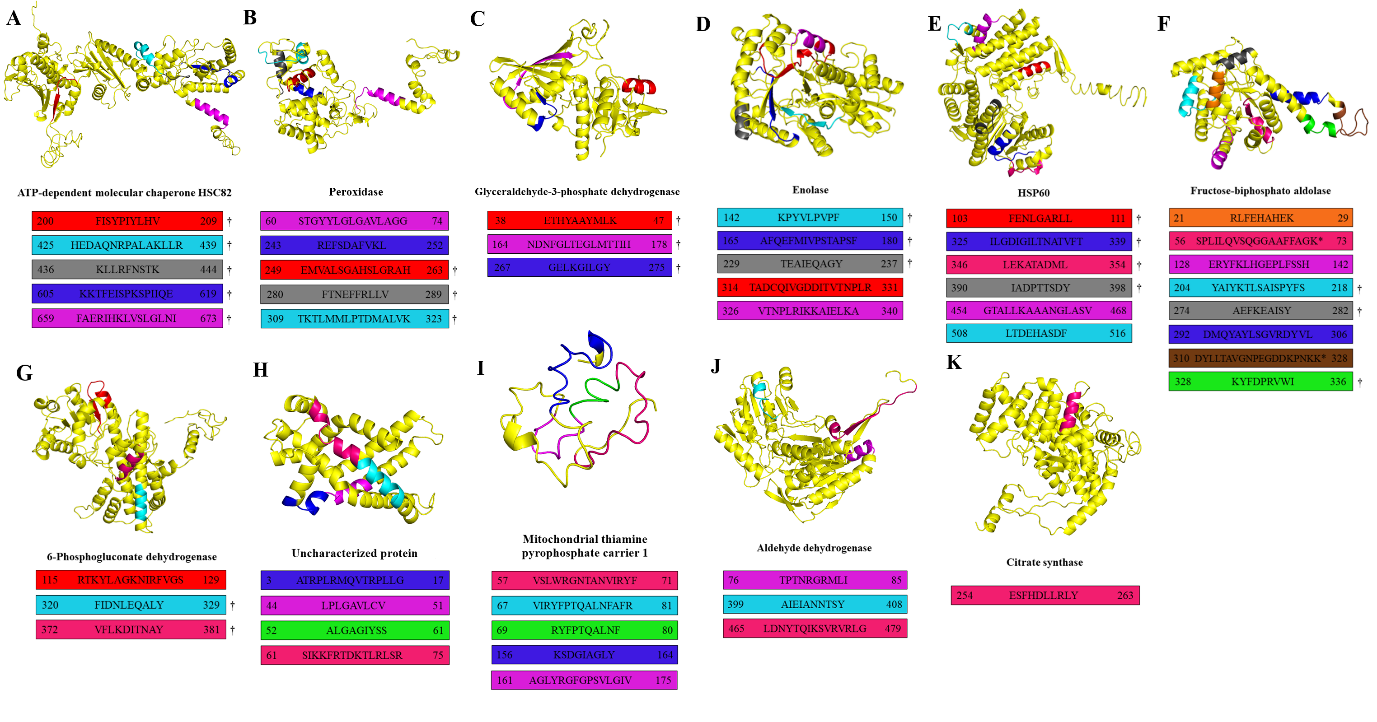


**Supplementary Figure 1. Representations of 3-D models of H. capsulatum proteins with exclusive and shared epitopes by other species of fungi.** Proteins with more than one epitope similar to other pathogenic fungi and not similar to human proteins were predicted by I-TASSER and refined by GalaxyRefine. Shared epitopes: **A.** HCAG_04686: ATP-dependent molecular chaperone HSC82, **B.** HCDG_01107: Peroxidase, **C.** HCEG_09258: Glyceraldehyde-3-phosphate dehydrogenase, **D.** HCEG_02034: Enolase, **E.** HCBG_08832: HSP-60 like protein, **F.** HCBG_06745: Fructose-bisphosphate aldolase, **G.** HCEG_08718: 6-Phosphogluconate dehydrogenase. (*) Sequences of peptides obtained through the method of immunoprecipitation that were naturally presented by BMDM and BMDCs of mice. (†) Sequences with 100% similarity with other species of fungi. Exclusive epitopes: **H.** HCDG_06427: Uncharacterized protein, **I.** HCDG_00690: Mitochondrial thiamine pyrophosphate carrier 1, **J.** HCAG_08367: Aldehyde dehydrogenase, **K.** HCEG_09064: Citrate synthase. Epitopes with 0% similarity with other species of pathogenic fungi.


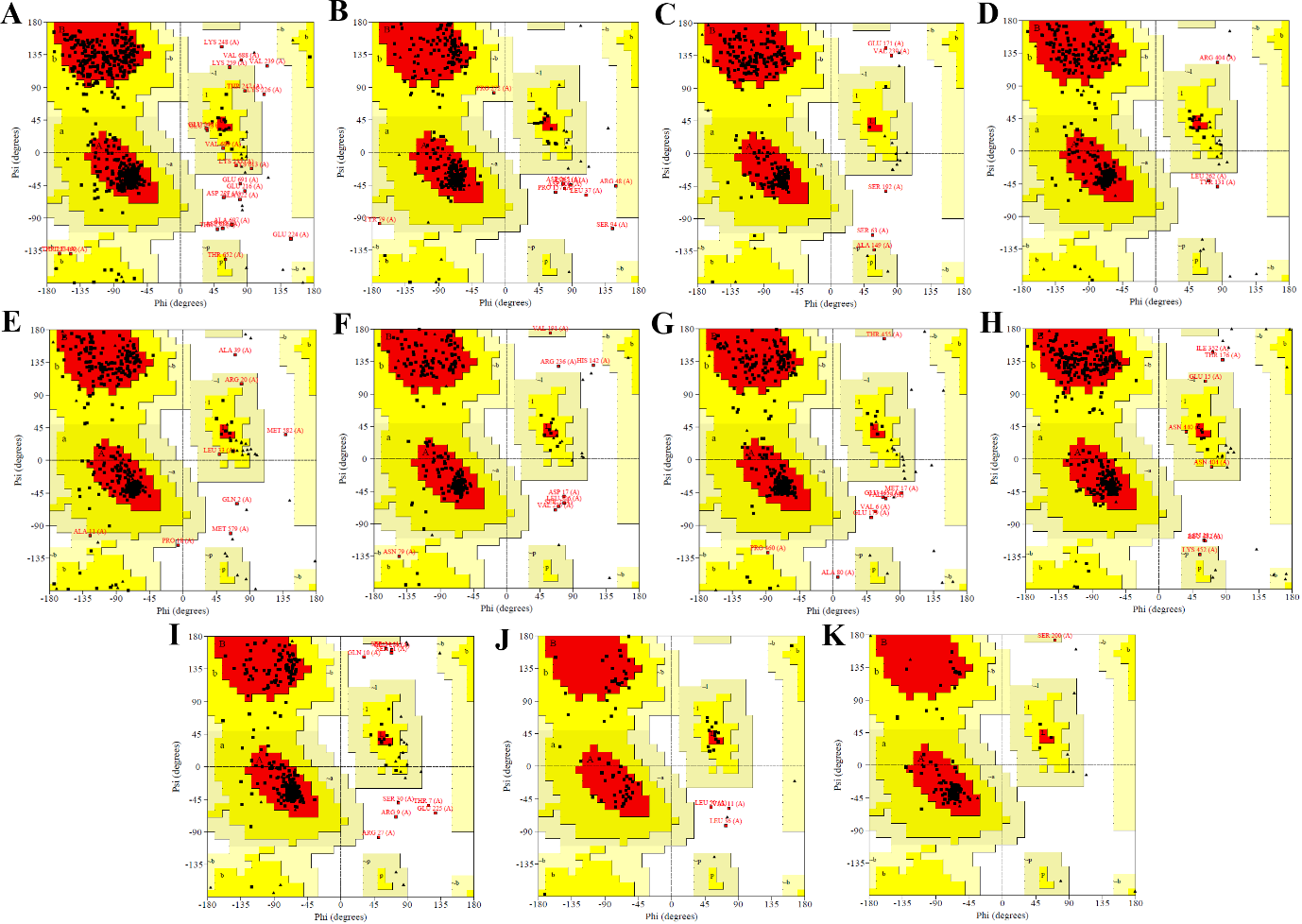


**Supplementary Figure 2. Validation of tertiary structures of shared and exclusive proteins of *H. capsulatum* by Ramachandran plots generated by PROCHECK server.** The numbers below and between parentheses refers to percentage of residues in most favoured regions [A,B,L], residues in additional allowed regions [a,b,l,p], residues in generously allowed regions [~a,~b,~l,~p] and residues in disallowed regions. **A.** HCAG_04686: ATP-dependent molecular chaperone HSC82 (87.8%, 8.8%, 1.5%, 1.8%); **B.** HCDG_01107: Peroxidase (87%, 10.8%, 0.3%, 1.9%); **C.** HCEG_09258: Glyceraldehyde-3-phosphate dehydrogenase (91.8%, 6.5%, 0.3%, 1.4%), **D.** HCEG_02034: Enolase (92.1%, 7.1%, 0.0%, 0.8%); **E.** HCBG_08832: HSP-60 like protein (91.3%, 7.3%, 0.6%, 0.8%); **F.** HCBG_06745: Fructose-bisphosphate aldolase (91.1%, 6.4%, 0.6%, 1.9%); **G.** HCEG_08718: 6-Phosphogluconate dehydrogenase (92.3%, 6.0%, 0.0%, 1.7%); **H.** HCAG_08367: Aldehyde dehydrogenase (92.4%, 5.7%, 0.9%, 0.9%); **I.** HCEG_09064: Citrate synthase (92.1%, 5.7%, 0.0%, 2.2%); **J.** HCDG_06427: Uncharacterized protein (67.6%, 27.9%, 0.0%, 4.4%); **K.** HCDG_00690: Mitochondrial thiamine pyrophosphate carrier 1 (94.3%, 5.2%, 0.5%, 0.0%).


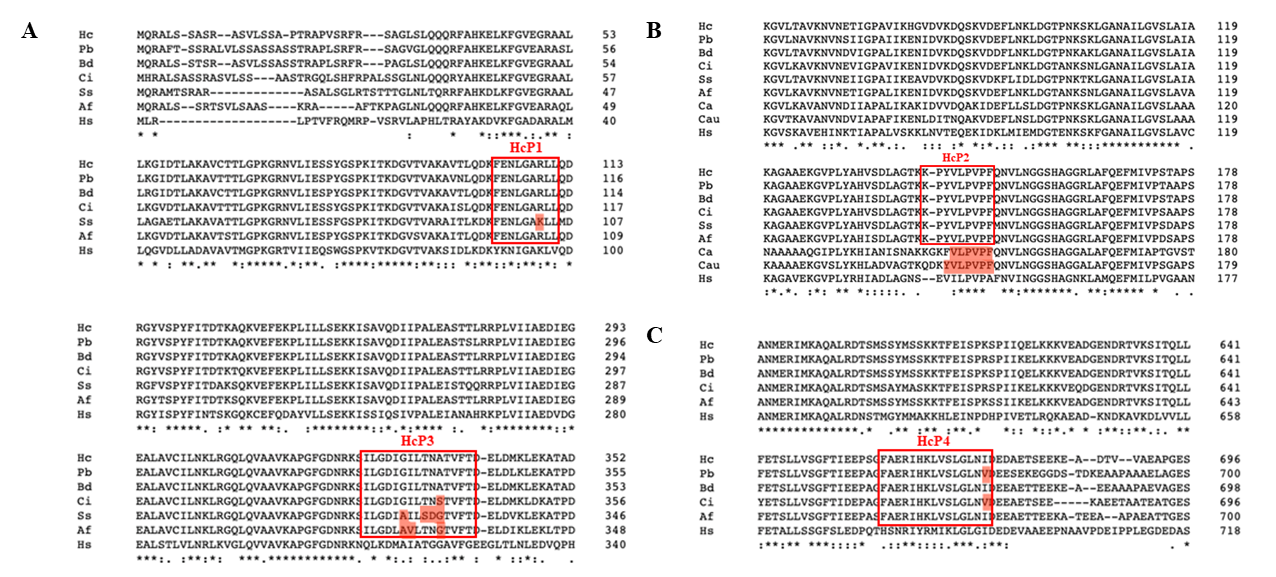


**Supplementary Figure 3. Alignment of candidate protein sequences. A.** HSP60, **B.** enolase, and **C.** HSC82 protein sequences of Hc: *Histoplasma capsulatum*, Pb: *Paracoccidioides brasiliensis*, Bd: *Blastomyces dermatitidis*, Ci: *Coccidioides immitis*, Ss: *Sporothrix schenckii*, Af: *Aspergillus fumigatus* and Hs: *Homo sapiens*. The sequences were aligned using Clustal Omega software. The regions of the fungi containing the peptide sequences synthesized from H. capsulatum were placed in a red box. Amino acids that differ from the sequence of H. capsulatum were highlighted in red inside of the box. * indicate equal sequences among all aligned species. : indicate amino acid difference for one or more species.


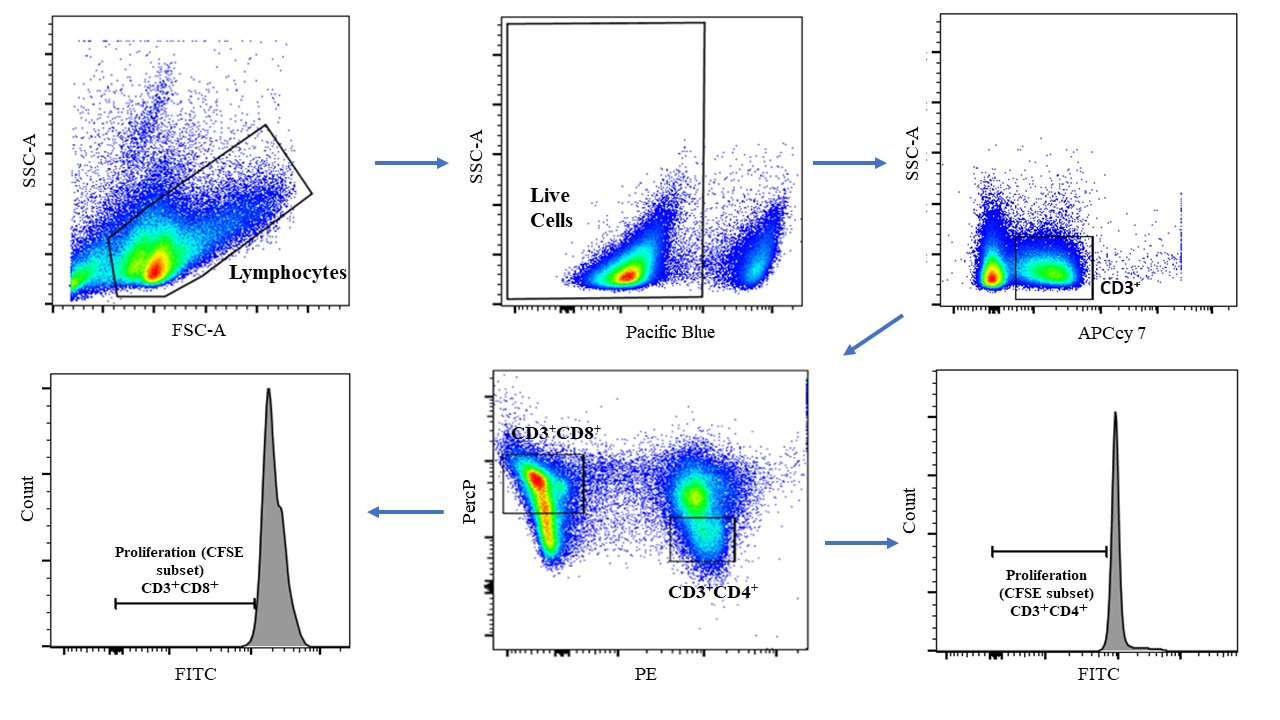


**Supplementary Figure 4. Analysis strategy used for proliferation of CD4+ and CD8+ T.** Cells were separated by granularity (SSC) and size (FSC), followed by live/dead cells singlet. Cells CD3+ were classified as lymphocytes. From that, the lymphocytes were classified in CD4+ and CD8+ and the proliferation of each population was analysed by the loss of CFSE fluorescence. The histogram shows the unstimulated cells control, which was used to compare the percentage of lymphoproliferation of the stimulated samples.

**2. Supplementary Tables**

**Supplementary Table 1.** Classification of *H. capsulatum* antigenic proteins resolved by 2-D SDS-PAGE immunoblotting for subcellular location, biological function, instability index (II), grand average of hydropathicity (GRAVY) molecular weight (MW, kDa: kilodaltons) and isoelectric point (pI).

| **Protein** | **Function** | **II** | **GRAVY** | **MW [kDa]** | **pI** | **Accession number^a^** |
| --- | --- | --- | --- | --- | --- | --- |
| Heat shock protein SSC1 | Stress response  Protein folding | 38.47 (stable) | -0.403 | 73.4 | 6.05 | HCBG_08743 |
| Hsp60-like protein | Protein refolding  ATP binding | 27.32 (stable) | -0.051 | 61.8 | 5.72 | HCBG_08832 |
| 4-Hydroxyphenylpyruvate dioxygenase | Fe(II)-dependent, non-heme oxygenase that catalyzes the conversion of 4-hydroxyphenylpyruvate to homogentisate | 39.20 (stable) | -0.328 | 45.0 | 5.77 | HCBG_03027 |
| Aha1_N | ATPase activator activity  Hsp90 protein binding | 36.60 (stable) | -0.605 | 36.5 | 5.62 | HCEG_02400 |
| Hsp70-like protein | ATP binding | 32.28 (stable) | -0.396 | 70.7 | 5.15 | HCBG_07920 |
| Succinate dehydrogenase flavoprotein subunit | It is involved in complex II of the mitochondrial electron transport chain and is responsible for transferring electrons from succinate to ubiquinone | 41.72 (unstable) | -0.444 | 71.1 | 6.83 | HCBG_06673 |
| Homogentisate 1,2-dioxygenase | Catalytic activity in the L-phenylalanine catabolic process and  Tyrosine metabolic process | 38.50 (stable) | -0.365 | 50.4 | 6.06 | HCAG_05721 |
| Aldehyde dehydrogenase | Oxidoreductase activity, acting on the aldehyde or oxo group of donors, NAD or NADP as acceptor | 23.96 (stable) | -0.048 | 52.7 | 6.11 | HCAG_08367 |
| 6-Phosphogluconate dehydrogenase | Catalyzes the oxidative decarboxylation of 6-phosphogluconate to ribulose 5-phosphate and CO2 | 33.30 (stable) | -0.239 | 54.1 | 6.48 | HCEG_08718 |
| Enolase | Phosphopyruvate hydratase activity in the Glycolytic process. Enzyme with two Mg2+ cofactors | 34.76 (stable) | -0.247 | 47.1 | 5.92 | HCEG_02034 |
| Fructose-bisphosphate aldolase | Catalyzes the aldol condensation of dihydroxyacetone phosphate with glyceraldehyde 3-phosphate to form fructose 1,6-bisphosphate in gluconeogenesis | 28.58 (stable) | -0.317 | 39.6 | 6.34 | HCBG_06745 |
| Elongation factor 1-gamma | Translation elongation factor activity | 38.86 (stable) | -0.417 | 45.0 | 8.10 | HCBG_08684 |
| ATP synthase subunit alpha | Produces ATP from ADP in the presence of a proton gradient across the membrane | 35.06 (stable) | -0.178 | 59.9 | 9.07 | HCBG_01891 |
| Citrate synthase | Transferase activity, transferring acyl groups, acyl groups converted into alkyl on transfer tricarboxylic acid cycle | 32.85 (stable) | -0.168 | 51.5 | 9.13 | HCEG_09064 |
| Alcohol dehydrogenase | oxidoreductase activity | 24.48  (Stable) | 0.167 | 37.1 | 7.99 | HCEG_08061 |
| Peroxidase | Response to oxidative stress. Peroxidase with Heme group (catalyzes reaction using iron ions present in the heme group). | 30.67 (stable) | -0.568 | 41.4 | 9.00 | HCDG_01107 |
| Malate dehydrogenase | Catalyzes the oxidation of malate to oxaloacetate in the tricarboxylic acid cycle | 30.26 (stable) | 0.007 | 35.7 | 9.26 | HCEG_05915 |

^a^ Access number to Uniprot database.

**Supplementary Table 2.** Classification of *H. capsulatum* antigenic proteins obtained by immunoprecipitation regarding subcellular location, biological function, instability index (II), grand average of hydropathicity (GRAVY) molecular weight (MW, kDa: kilodaltons) and isoelectric point (pI).

| **BLAST ID** | **Function** | **II** | **GRAVY** | **MW [kDa]** | **pI** | **Accession number^a^** |
| --- | --- | --- | --- | --- | --- | --- |
| Nascent polypeptide-associated complex subunit alpha | - | 47.94  (Unstable) | -0.596 | 22.0 | 4.86 | HCBG_02857 |
| Glyceraldehyde-3-phosphate dehydrogenase | involved in a subpathway of the glycolysis that synthesizes pyruvate from D-glyceraldehyde 3-phosphate | 28.81  (Stable) | -0.144 | 36.1 | 7.69 | HCEG_09258 |
| Pfs domain-containing protein | Catalytic activity in nucleoside metabolic process | 50.51  (Unstable) | -0.368 | 176.3 | 6.35 | HCBG_07657 |
| Ribosomal protein L37 | Structural constituent of ribosome | 48.06  (Unstable) | -1.364 | 10.5 | 11.40 | HCEG_00999 |
| Ribosomal protein L31e | Structural constituent of ribosome | 44.93  (Unstable) | -0.750 | 13.8 | 10.38 | HCAG_05192 |
| Ribosomal protein L7a | Ribosome biogenesis | 47.63  (Unstable) | -0.574 | 29.3 | 10.36 | HCAG_05221 |
| Ribosomal protein L14 | Structural constituent of ribosome | 47.78 (Unstable) | -0.516 | 24.7 | 10.24 | HCBG_02856 |
| 40S ribosomal protein S7 | Structural constituent of ribosome | 57.73  (Unstable) | -0.494 | 22.6 | 10.46 | HCEG_02939 |
| 40S ribosomal protein S5 | Structural constituent of ribosome and participate of the translation process in the RNA binding | 40.56  (Unstable) | -0.300 | 23.8 | 9.28 | HCEG_03546 |
| 40S ribosomal protein S7 | Structural constituent of ribosome | 55.22  (Unstable) | -0.193 | 9.6 | 8.16 | HCDG_03631 |
| 60S ribosomal protein L36 | Structural constituent of ribosome | 44.05  (Unstable) | -0.835 | 11.5 | 11.61 | HCBG_00063 |
| 60S ribosomal protein | Structural constituent of ribosome | 53.73  (Unstable) | -0.846 | 14.4 | 10.77 | HCEG_02183 |
| 60S ribosomal protein | Structural constituent of ribosome | 44.56  (Unstable) | -1.130 | 18.1 | 11.36 | HCEG_08256 |
| 60S ribosomal protein | Structural constituent of ribosome | 24.52  (Stable) | -0.489 | 16.9 | 10.39 | HCEG_04281 |
| 60S ribosomal protein L31 | Structural constituent of ribosome | 42.80  (Unstable) | -0.948 | 12.2 | 9.82 | HCDG_02034 |
| Uncharacterized protein | - | 34.52  (Stable) | -0.299 | 9.3 | 11.00 | HCDG_06427 |
| Uncharacterized protein | - | 47.73  (Unstable) | -0.462 | 30.0 | 9.73 | HCBG_06739 |
| Uncharacterized protein | - | 53.15  (Unstable) | -0.319 | 30.8 | 7.68 | HCAG_07106 |
| ATP-dependent molecular chaperone HSC82 | Protein folding and ATP binding | 39.38  (Stable) | -0.599 | 79.9 | 4.90 | HCAG_04686 |
| Lipase/serine esterase | NAD+ kinase activity | 46.22  (Unstable) | -0.539 | 156.8 | 6.78 | HCDG_00413 |
| Vacuolar protein sorting-associated protein 35 | Protein transport | 43.03  (Unstable) | -0.374 | 99.8 | 5.6 | HCEG_00302 |
| Serine hydroxymethyltransferase | involved in the pathway tetrahydrofolate interconversion (carbon metabolism) | 26.97  (Unstable) | -0.335 | 56.6 | 9.16 | HCAG_07418 |
| Isochorismatase domain-containing protein | Catalytic activity | 27.20  (Stable) | 0.198 | 20.8 | 8.56 | HCAG_07305 |
| Mitochondrial F1F0 ATP synthase subunit F Atp17 | ATP synthesis coupled proton transport | 41.36 (Unstable) | -0.238 | 11.2 | 10.13 | HCEG_06769 |
| 3-hydroxybutyryl CoA dehydrogenase | 3-hydroxyacyl-CoA dehydrogenase activity in the fatty acid metabolic process | 34.66  (Stable) | 0.065 | 34.2 | 7.64 | HCEG_04858 |
| Sm domain-containing protein | - | 64.66  (Stable) | -0.322 | 19.3 | 10.92 | HCBG_00112 |
| H15 domain-containing protein | Nucleosome assembly and DNA binding | 26.97  (Stable) | -0.924 | 20.8 | 10.81 | HCEG_07158 |
| Ribosomal protein S23 | Structural constituent of ribosome | 12.99  (Stable) | -0.559 | 15.7 | 10.48 | HCEG_08043 |
| Ribosomal protein L37a | Structural constituent of ribosome | 45.94  (Unstable) | -0.360 | 10.1 | 10.69 | HCEG_03019 |
| Ribosomal protein L14 | Structural constituent of ribosome and participate of the translation process in the RNA binding | 30.52  (Stable) | -0.399 | 16.4 | 10.49 | HCDG_01939 |
| 40S ribosomal protein S15 | Structural constituent of ribosome and participate of the translation process in the RNA binding | 31.22  (Stable) | -0.619 | 17.5 | 10.10 | HCBG_01774 |
| 60S ribosomal protein L2 | Structural constituent of ribosome | 28.16 (Stable) | -0.588 | 27.4 | 10.88 | HCDG_08240 |
| 60S ribosomal protein L13 | Structural constituent of ribosome | 48.41  (Unstable) | -0.637 | 24.3 | 11.40 | HCAG_07708 |
| 60S ribosomal protein L12 | Structural constituent of ribosome | 43.31  (Unstable) | -0.310 | 17.7 | 9.60 | HCDG_03181 |
| 60S ribosomal protein L27 | Structural constituent of ribosome | 33.15  (Stable) | -0.539 | 15.5 | 10.49 | HCEG_00238 |
| 60S ribosomal protein | Structural constituent of ribosome | 38.59  (Stable) | -0.360 | 40.1 | 10.90 | HCEG_06198 |
| 60S ribosomal protein | Structural constituent of ribosome | 41.51  (Unstable) | -0.479 | 21.1 | 11.81 | HCEG_01279 |
| 60s ribosomal protein | Structural constituent of ribosome | 54.21  (Unstable) | -0.218 | 16.5 | 9.61 | HCEG_08000 |
| Mitochondrial thiamine pyrophosphate carrier 1 | Transmembrane transporter activity | 11.91  (Stable) | -0.033 | 24.7 | 9.76 | HCDG_00690 |
| Uncharacterized protein | - | 63.64 (Unstable) | -0.954 | 33.3 | 10.53 | HCEG_00555 |
| Uncharacterized protein | - | 60.49  (Unstable) | -0530 | 11.9 | 10.53 | HCEG_05998 |

^a^ Access number to Uniprot database.

**Supplementary Table 3.** Characteristics of HLA-I restricted epitopes predicted *in silico* from 25 *H. capsulatum* antigenic proteins.

| **Protein^a^** | **Sequence** | **HS (%)^b^** | **Allele (%rank)^c^** |
| --- | --- | --- | --- |
| Aha1_N domain-containing protein (HCEG_02400) | DVVKRNWEGY | 0% | HLA-A2601 (0.08); HLA-A2602 (0.8); HLA-A2603 (0.6). |
|  | KELAKLAPAL | 0% | HLA-A0202 (1.3); HLA-A0205 (0.8); HLA-B4001 (0.3); HLA-B4002 (0.02); HLA-B4402 (0.4); HLA-B4403 (0.5); HLA-B4501 (0.6). |
| Aldehyde dehydrogenase (HCAG_08367) | TPTNRGRMLI | 0% | HLA-B0702 (0.7); HLA-B4201 (1.7); HLA-B5101 (1.8); HLA-B5301 (1.5); |
|  | AIEIANNTSY | 0% | HLA-A0101 (1.1); HLA-A2601 (0.8); HLA-A3002 (0.8); HLA-B1801 (0.6); HLA-B4402 (0.08); HLA-B4403 (0.04); HLA-B4501 (1.8). |
| ATP synthase  (HCBG_01891) | SPTEISSAL | 0% | HLA-A3215 (1.7); HLA-A6823 (1.7); HLA-B0702 (0.04); HLA-B1402 (1.2); HLA-B1509 (0.1); HLA-B3501 (0.12); HLA-B3503 (0.4); HLA-B3901 (0.09); HLA-B4201(0.05); HLA-B4801 (1.3); HLA-B5301 (0.8); HLA-B8301 (0.03); H-2-Kd (2); H-2-Ld (1). |
| ATP-dependent molecular chaperone HSC82 (HCAG_04686) | KLLRFNSTK | 0% | HLA-A0301 (0.01); HLA-A1101 (0.25); HLA-A3001 (0.17); HLA-A3101 (0.8); HLA-A3201 (0.8); HLA-B0802 (1.8). |
|  | FISYPIYLHV | 0% | HLA-A0201 (0.4); HLA-A0202 (0.3); HLA-A0203 (0.25); HLA-A0205 (0.4); HLA-A0206 (0.25); HLA-A0207 (0.9); HLA-A0217 (0.6); HLA-A6802 (0.15); HLA-B5101 (0.8); HLA-B5401 (1.1); H-2-Kb (0.5). |
| Citrate synthase (HCEG_09064) | ESFHDLLRLY | 0% | HLA-A2601 (0.17); HLA-A2602 (1.6); HLA-A2902 (0.5); HLA-A3002 (1.7). |
|  | GTEMLPEAMF | 0% | HLA-B4001 (1.6); HLA-B440 (0.03); HLA-B4403 (1.1); HLA-B5701 (1.9); HLA-B5802 (0.3). |
| Elongation factor 1-gamma (HCBG_08684) | KYPKELKLTF | 0% | HLA-A2301 (0.12); HLA-A2402 (0.01); HLA-A2403 (0.2); HLA-B0702 (0.9); HLA-B0801 (1.9); HLA-B0802 (1.2); HLA-B0803 (1.6); HLA-B5301 (0.6); HLA-B5802 (1.3); H-2-Dd (0.5). |
|  | SELVPTLGGW | 0% | HLA-A2602 (1.6); HLA-B4402 (0.04); HLA-B4403 (0.04). |
| Enolase  (HCEG_02034) | TEAIEQAGY | 0% | HLA-A2501 (1.4); HLA-A6823 (1.8); HLA-A8001 (1.6); HLA-B1801 (0.5); HLA-B4402 (0.02); HLA-B4403 (0.9). |
|  | YVLPVPFQNV | 0% | HLA-A0201 (0.4); HLA-A0203 (1.5); HLA-A0205 (0.2); HLA-A0206 (0.08); HLA-A0207 (0.03); HLA-A0217 (0.4); HLA-A6802 (1.5); HLA-A6901 (1.8). |
|  | KPYVLPVPF | 0% | HLA-A3201 (0.12); HLA-A3207 (1.7); HLA-A3215 (0.15); HLA-A6601 (1.1); HLA-A6823 (0.15); HLA-B0702 (0.5); HLA-B0802 (0.9); HLA-B0803(1.9); HLA-B1502 (2); HLA-B1503 (0.5); HLA-B1801 (2); HLA-B2720 (1.3); HLA-B3501 (0.17); HLA-B3503 (0.25); HLA-B3901 (1.8); HLA-B4013 (0.25); HLA-B4201 (0.08); HLA-B4601 (0.9); HLA-B4801 (1.1); HLA-B5101 (0.8); HLA-B5301 (0.3); HLA-B5401 (1.7); HLA-B8301 (0.07); H-2-Ld (0.05). |
|  | IVPSTAPSF | 0% | HLA-A0205 (1.8); HLA-A2402 (1.1); HLA-A2403 (1.4); HLA-A2601 (1.7); HLA-A2603 (1.6); HLA-A3201 (1.7); HLA-A3215 (1.7); HLA-A6823 (1.7); HLA-A2602 (1.1); HLA-B1501 (1.5); HLA-B1503 (0.7); HLA-B1517 (1.4); HLA-B3501 (1.4); H-2-Dd (1.4). |
| Fructose-biphosphate aldolase  (HCBG_06745) | ESVDNNSLY | 0% | HLA-A0101 (0.4); HLA-A2501 (0.06); HLA-A2601 (0.01); HLA-A2602 (0.03); HLA-A2603 (0.3); HLA-A2902 (1.2); HLA-A3002 (1.1); HLA-A6601 (1.2); HLA-A6801 (0.9); HLA-B1502 (1.9); HLA-B1517 (0.5); HLA-B3501 (1.2); HLA-B5802 (1.9). |
|  | AEFKEAISY | 0% | HLA-A3215 (1.4); HLA-A6823 (1.7); HLA-A8001 (0.4); HLA-B1501 (1.5); HLA-B1503 (0.3); HLA-B1801 (0.06); HLA-B4001 (0.9); HLA-B4002 (0.8); HLA-B4402 (0.01); HLA-B4403 (0.01); HLA-B4501 (0.8); HLA-B8301 (1.7). |
|  | LLAKHQAYV | 0% | HLA-A0201 (0.25); HLA-A0201 (1.5); HLA-A0202 (0.03); HLA-A0203 (0.03); HLA-A0205 (0.01); HLA-A0206 (0.7); HLA-A0207 (0.8); HLA-A0211 (0.2); HLA-A0212 (0.12); HLA-A0216 (0.08); HLA-A0217 (0.3); HLA-A0219 (0.03); HLA-A0250 (0.15); HLA-B0801 (1.6); HLA-B0802 (1); |
|  | RLFEHAHEK | 0% | HLA-A0211 (1.8); HLA-A0250 (0.8); HLA-A0301 (0.05); HLA-A1101 (0.3); HLA-A6823 (1.5); HLA-A6901 (0.6); HLA-B0803 (1); HLA-B2720 (1.2); HLA-B4013 (1.5). |
|  | KYLKRVTPMK | 0% | HLA-A0301 (0.06); HLA-A1101 (1.6); HLA-A2301 (2); HLA-A3001 (0.08); HLA-A3101 (0.9); H-2-Kd (0.7). |
|  | KYFDPRVWI | 0% | HLA-A2301 (0.6); HLA-A2402 (1.4); HLA-A2403 (0.25); HLA-A3001 (1.1); HLA-A3201 (1); HLA-A3207 (0.17); HLA-B4013 (0.17); HLA-B7301 (0.4); H-2-Kd (0.3). |
|  | TPMKQWLEM | 0% | HLA-A2602 (1.1); HLA-A3207 (1.6); HLA-A3215 (1.6); HLA-A6823 (0.8); HLA-A6901 (1.3); HLA-A8001 (1.5); HLA-B0702 (0.02); HLA-B0801 (0.4); HLA-B3501 (0.06); HLA-B3503 (0.17); HLA-B3901 (0.9); HLA-B4201 (0.15); HLA-B5101 (0.7); HLA-B5301 (0.17); HLA-B8301 (0.01); H-2-Ld (0.01). |
|  | SAISPYFSI | 0% | HLA-A0206 (1.3); HLA-A0217 (1.2); HLA-A2402 (1.6); HLA-A2403 (1.3); HLA-A2603 (1.5); HLA-A3201 (0.7); HLA-A3207 (0.15); HLA-A3215 (0.4); HLA-A6601 (1); HLA-A6802 (0.4); HLA-A6823 (1.8); HLA-A6901 (0.06); HLA-B0802 (2); HLA-B1503 (1.4); HLA-B1517 (1); HLA-B3901 (2); HLA-B4013 (0.6); HLA-B4801 (1.7); HLA-B5101 (0.9); HLA-B5301 (1.2); HLA-B5801 (0.9); HLA-B8301 (0.8); H-2-Db (0.12); H-2-Kb (0.3). |
| Glyceraldehyde-3-phosphate dehydrogenase  (HCEG_09258) | ETHYAAYMLK | 0% | HLA-A0301 (0.3); HLA-A1101 (0.4); HLA-A2601 (1.7); HLA-A3001 (1.2); HLA-A3301 (1.8); HLA-A6801 (0.07); HLA-A6802 (1.5). |
|  | GELKGILGY | 0% | HLA-A8001 (0.25); HLA-B1502(1.7); HLA-B1801 (0.2); HLA-B4001 (1.7); HLA-B4402 (0.25); HLA-B4403 (0.12). |
|  | KTYDPSVNV | 0% | HLA-A0201 (1.2); HLA-A0203 (1.3); HLA-A0206 (0.4); HLA-A0211 (0.25); HLA-A0212 (0.9); HLA-A0216 (0.7); HLA-A0219 (1.3); HLA-A0250 (1.2); HLA-A3001 (0.4); HLA-A3201 (0.25); HLA-A6601 (1.2); HLA-A6802 (0.4); HLA-A6823 (0.5); HLA-A6901 (0.07); HLA-B1503 (1.8); HLA-B1517 (0.4); HLA-B2720 (1.9); HLA-B4801 (1.5); HLA-B8301 (1.6); H-2-Kb (1.1). |
| Heat shock protein SSC1 (HCBG_08743) | GEVTDVLLL | 0% | HLA-B4001 (0.04); HLA-B4002 (0.9); HLA-B4402 (0.8); HLA-B4403 (0.5); HLA-B4801 (1.1); H-2-Kk (1.4). |
|  | TLFDKMHKA | 0% | HLA-A0201 (0.4); HLA-A0202 (0.6); HLA-A0203 (0.12); HLA-A0206 (1.8); HLA-A0211 (0.8); HLA-A0212 (0.4); HLA-A0216 (0.25); HLA-A0219 (0.4); HLA-A0250 (1.2); HLA-A2501 (1.9); HLA-A6901 (1.1). |
| Histone H15 (HCEG_07158) | RKAPVAPAV | 0% | HLA-A0205 (1.2); HLA-B2720 (0.07); HLA-B1503 (1.3); HLA-B1509 (0.4); HLA-B3901 (0.07); HLA-B4013 (0.7); HLA-B4801 (0.7); HLA-B8301 (1.5). |
|  | MIKDAIINL | 0% | HLA-A0202 (0.8); HLA-A0203 (0.6); HLA-A0206 (1.4); HLA-A0211 (1.2); HLA-A0212 (1.3); HLA-A0216 (2); HLA-A0217 (1.5); HLA-A0219 (1.8); HLA-A0250 (0.5); HLA-A2501 (1.9); HLA-A3001 (0.6); HLA-A3215 (1.9); HLA-A6802 (1.5); HLA-A6823 (1.5); HLA-A6901 (0.7); HLA-B0803 (0.3); HLA-B8301 (2); H-2-Ld (1). |
|  | TTKKAAAPK | 0% | HLA-A0301 (1.3); HLA-A1101 (0.6); HLA-A2603 (1.5); HLA-A3001 (0.04); HLA-A6601 (1.8); HLA-A6801 (0.4); HLA-B0802 (1); HLA-B0803 (0.9). |
| Homogentisate 1,2-dioxygenase (HCAG_05721) | YTYLHGFGSY | 0% | HLA-A0101 (0.12); HLA-A0301 (1); HLA-A1101 (1.2); HLA-A2501 (0.04); HLA-A2602 (1.1); HLA-A2603 (0.4); HLA-A2902 (0.03); HLA-A3002 (0.1); HLA-A3215 (0.6); HLA-A6823 (1); HLA-A8001 (0.8); HLA-B1501 (0.05); HLA-B1502 (0.9); HLA-B1503 (1.9); HLA-B1517 (0.9); HLA-B4601 (0.7). |
|  | SEAIDGALPV | 0% | HLA-A0206 (0.8); HLA-A6802 (1.7); HLA-A6901 (1.8); HLA-B4001 (0.25); HLA-B4002 (0.5); HLA-B4402 (0.7); HLA-B4403 (1.1); HLA-B4501 (0.3); HLA-B4601 (1); H-2-Kk (0.6). |
| HSP-60 (HCBG_08832) | IADPTTSDY | 0% | HLA-A0101 (0.05); HLA-A2902 (1.6); HLA-A3002 (0.6); HLA-A6601 (2); HLA-A6823 (2); HLA-A8001 (0.8); HLA-A2902 (1.6); HLA-A3002 (0.6); HLA-A6601 (2); HLA-A6823 (2); HLA-A8001 (0.8); HLA-B1502 (1.7); HLA-B1503 (1.6); HLA-B3501 (0.12); HLA-B4601 (1.7); HLA-B5801 (1.6). |
|  | FENLGARLL | 0% | HLA-B1509 (0.5); HLA-B1801 (0.6); HLA-B2720 (1.7); HLA-B3503 (1.2); HLA-B3901 (0.9); HLA-B4001 (0.09); HLA-B4002 (0.15); HLA-B4013 (1.4); HLA-B4801 (0.8); HLA-B4403 (1.9); HLA-B4402 (0.6); H-2-Kk (0.01). |
|  | LEKATADML | 0% | HLA-B4001 (0.17); HLA-B4002 (0.9); HLA-B4801 (1.8); H-2-Kk (0.7). |
|  | FTDELDMKL | 0% | HLA-A0101 (0.12); HLA-A0201 (1.0); HLA-A0202 (1.6); HLA-A0206 (0.4); HLA-A0211 (1.4); HLA-A0212 (0.6); HLA-A0216 (0.4); HLA-A0217 (0.8); HLA-A2501 (1.8); HLA-A6823 (0.6); HLA-A6901 (0.5); HLA-B0803 (1.8); HLA-B1509 (0.7); HLA-B1517 (0.8); HLA-B3801 (1.7); HLA-B3901 (1.1); HLA-B5301 (1.5). |
|  | LTDEHASDF | 0% | HLA-A0101 (0.1); HLA-A2602 (0.7); HLA-B0803 (0.8); HLA-B1503 (1.6); HLA-B1517 (0.6); HLA-B3501 (1.5); HLA-B5802 (1.5). |
| Hsp70-like protein (HCBG_07920) | KSEIDKTVQW | 0% | HLA-B4402 (0.15); HLA-B4403 (0.25); HLA-B5701 (0.17); HLA-B5801 (0.05); HLA-B5802 (0.2). |
|  | VANPIMMKF | 0% | HLA-A3201 (2); HLA-A3215 (1); HLA-A6601 (0.4); HLA-A6823 (1.9); HLA-A8001 (1.6); HLA-B1503 (1.4); HLA-B1517 (0.5); HLA-B3501 (1.3); HLA-B4601 (0.12); HLA-B5101 (1.1); HLA-B5301 (0.3); HLA-B5701 (0.12); HLA-B5801 (0.08); HLA-B5802 (0.4); HLA-B8301 (0.6). |
| Peroxidase  (HCDG_01107) | REFSDAFVKL | 0% | HLA-A2402; HLA-B4001 (0.07); HLA-B4002 (0.03); HLA-B4013 (1.5); HLA-B4402 (1.4); HLA-B4403 (0.5). |
|  | MMLPTDMALV | 0% | HLA-A0201 (0.12); HLA-A0202 (0.25); HLA-A0203 (0.3); HLA-A0205 (0.9); HLA-A0206 (0.09); HLA-A0207 (0.2); HLA-A0217 (0.02); HLA-A0250 (1.6); HLA-A2902 (1.8); HLA-A6802 (1.7). |
|  | FTNEFFRLLV | 0% | HLA-A0101 (0.25); HLA-A0201 (0.9); HLA-A0202 (0.8); HLA-A0203 (1); HLA-A0206 (0.25); HLA-A0207 (1.6); HLA-A0217 (1.1); HLA-A2601 (1.3); HLA-A6802 (0.5). |
| Ribosomal protein L14  (HCDG_01939) | LSHATLTPF | 0% | HLA-A2301 (1.9); HLA-A2601 (1.4); HLA-A2602 (0.5); HLA-A3201 (0.25); HLA-A3207 (1.3); HLA-A3215 (0.25); HLA-A6601 (0.09); HLA-A6823 (0.7); HLA-B0803 (2); HLA-B1402 (0.25); HLA-B1501 (0.01); HLA-B1502 (0.4); HLA-B1503 (0.03); HLA-B1517 (0.03); HLA-B3501 (0.3); HLA-B3503 (2); HLA-B4601 (0.4); HLA-B5701 (0.4); HLA-B5801 (0.4); HLA-B8301 (1.9); H-2-Db (1.3); H-2-Kb (1.8); H-2-Ld (0.8). |
|  | RVVLIRSGPY | 0% | HLA-A0301 (1.5); HLA-A2601 (1.5); HLA-A2902 (1.3); HLA-A3002 (0.3); HLA-A8001 (0.9); HLA-B1501 (0.5). |
| Sm protein  (HCBG_00112) | RPAGRGLPV | 0% | HLA-A6601 (0.5); HLA-B0702 (0.01); HLA-B0801 (1.3); HLA-B0802 (1.9); HLA-B1402 (0.9); HLA-B2720 (0.4); HLA-B3901 (1.3); HLA-B4013 (0.3); HLA-B4201 (0.7); HLA-B5401 (1.5); HLA-B8301 (0.01). |
|  | KMHMNLVLA | 0% | HLA-A0201 (1.5); HLA-A0203 (0.9); HLA-A0211 (0.5); HLA-A0212 (0.5); HLA-A0216 (1.6); HLA-A0219 (1.8); HLA-A0250 (0.6); HLA-A3001 (0.09); HLA-A3201 (1.7); HLA-B1503 (1.2); HLA-B4801 (0.7); H-2-Db (0.17). |
| Mitochondrial thiamine pyrophosphate carrier 1 (HCDG_00690) | KSDGIAGLY | 0% | HLA-A0101 (0.01); HLA-A2902 (0.9); HLA-A3002 (0.01); HLA-A8001 (0.05); HLA-B1517 (0.8); HLA-B5801 (0.4); HLA-B5802 (0.8). |
|  | RYFPTQALNF | 0% | HLA-A2301 (0.01); HLA-A2402 (0.01); HLA-A2403 (0.3); HLA-A2902 (1); HLA-A3002 (1.3); H-2-Dd (1.3). |
|  | MYDSIKPVL | 0% | HLA-A2301 (0.8); HLA-A2402 (1.7); HLA-A2403 (0.4); HLA-A3207 (1.7); HLA-A3215 (1.7); HLA-B1509 (0.3); HLA-B3901 (0.5); HLA-B4801 (0.9). |
| Uncharacterized protein (HCDG_06427) | KENQSAHTI | 0% | HLA-A3201 (0.8); HLA-B0803 (1.8); HLA-B2720 (1.3); HLA-B3901 (1.5); HLA-B4001 (0.15); HLA-B4002 (0.05); HLA-B4013 (1.1); HLA-B4402 (0.04); HLA-B4403 (0.12); HLA-B4501 (1); HLA-B8301 (1.5); H-2-Kd (0.4); H-2-Kk (0.03). |
|  | LPLGAVLCV | 0% | HLA-A0216 (2); HLA-A6901 (0.9); HLA-B0702 (1.3); HLA-B3501 (1.2); HLA-B3503 (1.5); HLA-B3901 (1.4); HLA-B5101 (0.07); HLA-B5301 (0.4); HLA-B5401 (0.4); HLA-B8301 (0.5); H-2-Ld (0.5). |
|  | ALGAGIYSS | 0% | HLA-A0211 (0.4); HLA-A0212 (0.7); HLA-A0216 (0.25); HLA-A0219 (0.6); HLA-A0250 (0.5). |
| 4-Hydroxyphenylpyruvate dioxygenase (HCBG_03027) | EYYEKVLGF | 0% | HLA-A2301 (0.5); HLA-A2402 (0.4); HLA-A2403 (0.5); HLA-A2501 (0.6); HLA-A2601 (0.8); HLA-A2602 (0.4); HLA-A2603 (0.15); HLA-A3207 (0.2); HLA-A3215 (0.5); HLA-A6601 (0.4); HLA-A6823 (1.7); HLA-B0802 (0.5); HLA-B0803 (2); HLA-B1402 (0.25); HLA-B1502 (1.4). |
| 6-Phosphogluconate dehydrogenase (HCBG_03027) | FIDNLEQALY | 0% | HLA-A0101 (0.02); HLA-A2601 (1.2); HLA-A290 (0.7); HLA-A3002 (1.4); HLA-B3501 (1.1); HLA-B4402 (1.9). |
|  | VFLKDITNAY | 0% | HLA-A2501 (1.7); HLA-A2601 (1.4); HLA-A2902 (0.4); HLA-A3002 (0.7); HLA-B1501 (0.8); HLA-B1502 (0.9); HLA-B4601 (0.06). |
| 40S ribosomal protein S15  (HCBG_01774) | VPEMIGSII | 0% | HLA-B0702 (0.6); HLA-B3503 (2); HLA-B4201 (1); HLA-B5101 (0.09); HLA-B5301 (0.8); HLA-B5401 (2); HLA-B8301 (0.4); H-2-Ld (0.6). |
| 60S ribosomal protein (HCEG_04281) | RTSTTFHRPK | 0% | HLA-A0301 (0.2); HLA-A1101 (0.07); HLA-A3001 (0.3); HLA-A3101 (0.3); HLA-A6801 (2). |
|  | HPLNTESAM | 0% | HLA-A2602 (1.6); HLA-A2603 (1.2); HLA-A3207 (1.8); HLA-A3215 (1.6); HLA-A6823 (1.8); HLA-B0702 (0.03); HLA-B1502 (0.5); HLA-B1509 (0.1); HLA-B3501 (0.01); HLA-B3503 (0.01); HLA-B3901 (0.4); HLA-B4201 (0.2); HLA-B5301 (0.17); HLA-B5401 (1.6); HLA-B8301 (0.05); H-2-Ld (0.9). |
| 60S ribosomal protein L2  (HCDG_08240) | ETFIANEGMY | 0% | HLA-A0101 (0.5); HLA-A2501 (0.25); HLA-A2601 (0.03); HLA-A2602 (0.8); HLA-A2603 (1.9); HLA-A2902 (0.25); HLA-A3002 (0.4); HLA-A6801 (0.7). |
|  | KYKDVVETF | 0% | HLA-A2301 (0.05); HLA-A2402 (0.4); HLA-A2403 (0.03); HLA-A3201 (1.3); HLA-A3207 (0.4); HLA-B0802 (1.5); HLA-B0803 (0.2); HLA-B1503 (1.6); HLA-B4601 (0.6); HLA-B5802 (0.06); HLA-B8301 (1.5); H-2-Kd (1.2). |
|  | FIYAGKNATL | 0% | HLA-A0201 (1.3); HLA-A0202 (0.8); HLA-A0203 (0.8); HLA-A0206 (1.6); HLA-A0217 (1.1); HLA-A2301 (2); HLA-A2402 (0.5); HLA-B0801 (1); HLA-B1501 (1.7); HLA-B1502 (1.5); HLA-B3503 (1.2); H-2-Kd (1.7). |
| 60S ribosomal protein L27  (HCEG_00238) | ITRGRYAGK | 0% | HLA-A0301 (0.25); HLA-A2501 (1.7); HLA-A2603 (0.6); HLA-A3001 (0.02); HLA-A3101 (0.7); HLA-A3301 (1.8); HLA-B0802 (0.5); HLA-B8301 (1.8). |
|  | KAHPFPYAL | 0% | HLA-A2403 (2); HLA-A3001 (0.4); HLA-A3201 (0.3); HLA-A3207 (0.1); HLA-A3215 (0.4); HLA-A6823 (0.3); HLA-B0702 (1.7); HLA-B0802 (1.1); HLA-B1503 (0.6); HLA-B1509 (1.4); HLA-B1517 (1.1); HLA-B2720 (0.6); HLA-B3501 (1); HLA-B3901 (0.3); HLA-B4013 (0.1); HLA-B4201 (0.8); HLA-B4601 (1.2); HLA-B4801 (0.08); HLA-B5701 (0.6); HLA-B5801 (1.1); HLA-B8301 (1.1); H-2-Db (1.6); H-2-Kb (0.7); H-2-Ld (0.25). |

^a^ Protein: Protein name and Access number to Uniprot database.

^b^ HS (%) - Human Similarity: Percentage of similarity of the peptide sequence predicted as T cell epitope with the human protein sequence.

^c^ Allele (%rank): The values shown indicate strong binders (SB) when % < 0.5 and weak binders (WB) when % < 2.

**Supplementary Table 4.** Characteristics of HLA-II restricted epitopes predicted *in silico* and obtained by immunoprecitation (marked with ^*^) from 26 antigenic proteins of *H. capsulatum.*

| **Protein^a^** | **Sequence** | **HS (%)^b^** | **Allele (IC50 nM)^c^** |
| --- | --- | --- | --- |
| Aha1_N domain-containing protein (HCEG_02400) | RKELAKLAPALIAEH | 0% | HLA-DRB10101 (5.1); HLA-DRB10401 (403.7); HLA-DRB10404 (328.2); HLA-DRB10701 (92.1); HLA-DRB10802 (185.2); HLA-DRB10901 (14); HLA-DRB11101 (110.4); HLA-DRB11302 (279); HLA-DRB11501 (185.7); HLA-DRB40101 (254); HLA-DRB50101 (109); HLA-DPA10201-DPB10101 (249.8); HLA-DQA10102-DQB10602 (103.7); HLA-DQA10401-DQB10402 (341.3); HLA-DQA10501-DQB10301 (56.8). |
|  | GEYIKLESPTSITQK | 0% | HLA-DRB10101 (5.3); HLA-DRB10301 (382.9); HLA-DRB10401 (10.8); HLA-DRB10404 (70.9); HLA-DRB10405 (37.5); HLA-DRB10701 (100.2); HLA-DRB10802 (186.9); HLA-DRB10901 (16.9); HLA-DRB11101 (135.7); HLA-DRB11302 (17.4); HLA-DRB11501 (359.6); HLA-DRB30101 (154); HLA-DRB40101 (205.3); HLA-DRB50101 (72.5). |
| Aldehyde dehydrogenase (HCAG_08367) | LSGFGRTAGAAIAAH | 0% | HLA-DRB10101 (6.9); HLA-DRB10401 (368.5); HLA-DRB10701 (13.9); HLA-DRB10901 (16.1); HLA-DRB11101 (273.3); HLA-DRB11501 (399.7); HLA-DRB50101 (116.9); HLA-DQA10102-DQB10602 (33); HLA-DQA10401-DQB10402 (217.5); HLA-DQA10501-DQB10301 (4.8); H-2-IAb (233.2). |
|  | LDNYTQIKSVRVRLG | 0% | HLA-DRB10101 (3.7); HLA-DRB10301 (422.1); HLA-DRB10401 (29.8); HLA-DRB10404 (125.5); HLA-DRB10405 (96.2); HLA-DRB10701 (4.3); HLA-DRB10802 (226.2); HLA-DRB10901 (71.4); HLA-DRB11101 (38.8); HLA-DRB11302 (244.4); HLA-DRB11501 (35); HLA-DRB40101 (173.5); HLA-DRB50101 (15.8); HLA-DPA10201-DPB10101 (339.1); HLA-DQA10102-DQB10602 (126.6); HLA-DQA10501-DQB10301 (382); H-2-IAd (350.8). |
| ATP synthase  (HCBG_01891) | IVGARAAAPGPLSAA | 0% | HLA-DRB10101 (29.2); HLA-DRB10802 (265.3); HLA-DRB10901 (394.7); HLA-DRB11302 (199.5); HLA-DQA10501-DQB10301 (4.3); H-2-IAb (47.2); H-2-IAd (435.8). |
|  | GRIVGARAAAPGPLS | 0% | HLA-DRB10101 (24.5); HLA-DRB10401 (368.8); HLA-DRB10802 (53.3); HLA-DRB10901 (344); HLA-DRB11101 (126); HLA-DRB11302 (156.2); HLA-DRB50101 (249.9); HLA-DQA10102-DQB10602 (191.2); HLA-DQA10501-DQB10301 (4.4); H-2-IAb (63.4). |
| ATP-dependent molecular chaperone HSC82 (HCAG_04686) | QMYYITGESLKAVQK | 66% | HLA-DRB10101 (4.8); HLA-DRB10401 (40.7); HLA-DRB10404 (391.2); HLA-DRB10405 (97.2); HLA-DRB10701 (67.6); HLA-DRB10802 (402.5); HLA-DRB10901 (34.8); HLA-DRB11101 (84.2); HLA-DRB11501 (94.6); HLA-DRB40101 (196.6); HLA-DRB50101 (36); HLA-DPA10103-DPB10401 (363.7); HLA-DPA10103-DPB10201 (179.5); HLA-DPA10201-DPB10101 (64); LA-DPA10201-DPB10501 (190.9); HLA-DPA10103-HLA-DPB10301_DPB10401 (138.4); HLA-DPA10301-DPB10402 (11.2); HLA-DQA10102-DQB10602 (420.7); HLA-DQA10501-DQB10301 (73.9). |
|  | FAERIHKLVSLGLNI | 42% | HLA-DRB10101 (5.4); HLA-DRB10401 (123.7); HLA-DRB10404 (205.7); HLA-DRB10405 (67.3); HLA-DRB10701 (4.9); HLA-DRB10901 (97.5); HLA-DRB11101 (34.5); HLA-DRB11501 (30.1); HLA-DRB40101 (26.1); HLA-DRB50101 (64.3); HLA-DPA10201-DPB10101 (340.7); HLA-DPA10301-DPB10402 (136.3). |
|  | HEDAQNRPALAKLLR | 60% | HLA-DRB10101 (36.3); HLA-DRB50101 (179.4); HLA-DQA10102-DQB10602 (422.4); HLA-DQA10501-DQB10301 (136.7); H-2-IAd (409.5). |
|  | KKTFEISPKSPIIQE | 50% | HLA-DRB10101 (168); HLA-DRB10301 (255.6); HLA-DRB10701 (28.2); HLA-DRB10901 (414.9); HLA-DRB11101 (157.1); HLA-DRB11302 (320.7); HLA-DRB30101 (298.9); HLA-DRB50101 (425.5); HLA-DPA10201-DPB10101 (482.6); H-2-IAb (292.2). |
| Citrate synthase (HCEG_09064) | QVRAFSRELAEKSLL | 0% | HLA-DRB10101 (10.1); HLA-DRB10301 (317.1); HLA-DRB10401 (90.9); HLA-DRB10405 (479.5); HLA-DRB10901 (158.4); HLA-DRB11101 (134.3); HLA-DRB11501 (457.4); HLA-DRB50101 (148); HLA-DPA10201-DPB10101 (239.2). |
| Elongation factor 1-gamma (HCBG_08684) | IAIYLASQNEKTTLL | 0% | HLA-DRB10101 (4.9); HLA-DRB10401 (52.3); HLA-DRB10404 (25.5); HLA-DRB10405 (74.3); HLA-DRB10701 (69.2); HLA-DRB10901 (283.4); HLA-DRB11101 (308.9); HLA-DRB11501 (236.8); HLA-DRB40101 (233.3); HLA-DRB50101 (79); HLA-DPA10201-DPB10101 (290.3); HLA-DQA10501-DQB10301 (239.7); |
|  | GGFFTRLEASRKYVF | 50% | HLA-DRB10101 (4.1); HLA-DRB10301 (44.2); HLA-DRB10401 (20.5); HLA-DRB10404 (208.2); HLA-DRB10405 (64.4); HLA-DRB10701 (119.1); HLA-DRB10802 (214.4); HLA-DRB10901 (19.8); HLA-DRB11101 (39.1); HLA-DRB11501 (192.1); HLA-DRB30101 (234.7); HLA-DRB50101 (5.1); HLA-DPA10201-DPB10501 (274.4); HLA-DQA10102-DQB10602 (293.5); HLA-DQA10501-DQB10301 (292.6). |
| Enolase  (HCEG_02034) | TADCQIVGDDITVTNPLR^*^ | 66% | HLA-DRB10301 (59.5); HLA-DRB10401 (218.3); HLA-DRB10701 (248.1); HLA-DRB10802 (458.3); HLA-DRB30101 (33.4); HLA-DPA10301-DPB10402 (394.5); HLA-DQA10501-DQB10301 (297.5). |
|  | QLADLYKQLASKYPI | 60% | HLA-DRB10101 (15.8); HLA-DRB10401 (101.9); HLA-DRB10404 (220.6); HLA-DRB10405 (187.6); HLA-DRB10701 (88); HLA-DRB10901 (61.7); HLA-DRB11101 (44.4); HLA-DRB11501 (117.1); HLA-DRB40101 (123.1); HLA-DRB50101 (14.3); HLA-DPA10201-DPB10101 (367.1). |
|  | VTNPLRIKKAIELKA | 60% | HLA-DRB10101 (40.6); HLA-DRB10701 (112.2); HLA-DRB10802 (332.5); HLA-DRB10901 (264.6); HLA-DRB11101 (115.4); HLA-DRB11302 (53.7); HLA-DRB11501 (397.4); HLA-DRB40101 (96.4); HLA-DRB50101 (105.8); H-2-IAd (295.6). |
|  | RIKKAIELKACNALL | 60% | HLA-DRB10101 (35.6); HLA-DRB10404 (232); HLA-DRB10701 (443.2); HLA-DRB10802 (339.9); HLA-DRB10901 (189.5); HLA-DRB11101 (264.1); HLA-DRB11302 (123.8); HLA-DRB11501 (469.3); HLA-DRB40101 (235.1); HLA-DRB50101 (441.4); HLA-DPA10201-DPB10501 (439.8); H-2-IAd (366.3). |
|  | AFQEFMIVPSTAPSF | 60% | HLA-DRB10101 (6.9); HLA-DRB10401 (41.7); HLA-DRB10404 (21.5); HLA-DRB10405 (35); HLA-DRB10701 (21.2); HLA-DRB10802 (119.2); HLA-DRB10901 (116.7); HLA-DRB11101 (153.9); HLA-DRB11302 (44.2); HLA-DRB30101 (156.4); HLA-DRB50101 (188.1); HLA-DPA10103-DPB10401 (323); HLA-DPA10201-DPB10101 (152.6); HLA-DPA10103-HLA-DPB10301_DPB10401 (346.7); HLA-DPA10301-DPB10402 (276); H-2-IAb (167.7). |
|  | EFMIVPSTAPSFSEA | 60% | HLA-DRB10101 (10); HLA-DRB10401 (71.3); HLA-DRB10404 (26.9); HLA-DRB10405 (237.4); HLA-DRB10701 (54.4); HLA-DRB10802 (54.1); LA-DRB10901 (71.7); HLA-DRB11101 (182.5); HLA-DRB11302 (22.6); LA-DRB30101 (99.1); HLA-DRB50101 (246.7); H-2-IAb (119.5). |
| Fructose-biphosphato aldolase (HCBG_06745) | VNLDTDMQYAYLSGVR^*^ | 0% | HLA-DRB10101 (99.8); HLA-DRB10301 (56.7); HLA-DRB10401 (460.3); HLA-DRB10901 (452.2); HLA-DRB11501 (119.8); HLA-DRB30101 (68); HLA-DPA10103-DPB10401 (284.6); HLA-DPA10103-DPB10201 (246.6); HLA-DPA10201-DPB10101 (230.1); HLA-DPB10301_DPB10401 (131.1); HLA-DPA10301-DPB10402 (213.4); HLA-DQA10102-DQB10602 (141.2); HLA-DQA10501-DQB10301 (84.3). |
|  | VQEGLDDFNTSGQL^*^ | 0% | Binding affinity above 500 nm |
|  | DYLLTAVGNPEGDDKPNKK^*^ | 0% | HLA-DRB10404 (27.6); HLA-DRB10405 (54.1); HLA-DRB10802 (419.1); HLA-DQA10501-DQB10301 (466.3). |
|  | GYAIPAINVTSSSTVVAALEAAR^*^ | 0% | HLA-DRB10101 (13.1); HLA-DRB10401 (123.8); HLA-DRB10404 (22); HLA-DRB10405 (143.3); HLA-DRB10701 (8.6); HLA-DRB10802 (21.7); HLA-DRB10901 (22.6); HLA-DRB11302 (26); HLA-DRB11501 (419.6); HLA-DRB40101 (323.8); HLA-DRB50101 (269.8); HLA-DQA10102-DQB10602 (11.9); HLA-DQA10301-DQB10302 (37.4); HLA-DQA10401-DQB10402 (68.7); HLA-DQA10501-DQB10201 (347.2); HLA-DQA10501-DQB10301 (11.6); H-2-IAb (192.8). |
|  | SPLILQVSQGGAAFFAGK^*^ | 0% | HLA-DRB10101 (34.1); HLA-DRB10401 (174.8); HLA-DRB10404 (90.3); HLA-DRB10701 (316.6); HLA-DRB10802 (352.7); HLA-DRB10901 (50.9); HLA-DRB11302 (27.1); HLA-DRB11501 (188.5); HLA-DRB40101 (20.2); HLA-DQA10102-DQB10602 (182.5); HLA-DQA10501-DQB10301 (9.4). |
|  | KTGVIVGDDVLR^*^ | 0% | HLA-DRB10301 (191.1); HLA-DQA10501-DQB10201 (398.1). |
|  | YAIYKTLSAISPYFS | 0% | HLA-DRB10101 (4); HLA-DRB10401 (21.8); HLA-DRB10404 (28); HLA-DRB10405 (26.9); HLA-DRB10701 (32.5); HLA-DRB10802 (129.4); HLA-DRB10901 (24.9); HLA-DRB11101 (19); HLA-DRB11501 (33.5); HLA-DRB50101 (35.1); HLA-DPA10201-DPB10101 (382.3); HLA-DQA10102-DQB10602 (192.8); HLA-DQA10501-DQB10301 (81.9); H-2-IAb (253.3). |
|  | DMQYAYLSGVRDYVL | 0% | HLA-DRB10101 (4.1); HLA-DRB10401 (85.7); HLA-DRB10404 (144); HLA-DRB10405 (31.4); HLA-DRB10701 (54.8); HLA-DRB10901 (215.8); HLA-DRB11101 (55.4); HLA-DRB11501 (31.4); HLA-DRB50101 (30.9); HLA-DPA10103-DPB10401 (401); HLA-DPA10103-DPB10201 (342.4); HLA-DPA10201-DPB10101 (115.4); HLA-DPA10103-HLA-DPB10301_DPB10401 (247.2); HLA-DPA10301-DPB10402 (173.3); HLA-DQA10101-DQB10501 (308.1); HLA-DQA10501-DQB10301 (93.3). |
|  | ERYFKLHGEPLFSSH | 0% | HLA-DRB10101 (4.3); HLA-DRB10401 (212.8); HLA-DRB10405 (389.5); HLA-DRB10701 (12.9); HLA-DRB10901 (77.3); HLA-DRB11101 (200.7); HLA-DRB11501 (105.3); HLA-DRB50101 (82); HLA-DPA10201-DPB10101 (252.7); HLA-DPA10301-DPB10402 (288.7); HLA-DQA10102-DQB10602 (444.7); HLA-DQA10501-DQB10201 (449.6); HLA-DQA10501-DQB10301 (65.5). |
|  | QEASIAGATAAAHYI | 0% | HLA-DRB10101 (49.3); HLA-DRB10401 (189.3); HLA-DRB10701 (251.2); HLA-DRB10802 (374.8); HLA-DRB10901 (453.7); HLA-DRB50101 (45.5); HLA-DQA10102-DQB10602 (32.7); HLA-DQA10301-DQB10302 (406.7); HLA-DQA10401-DQB10402 (220.5); HLA-DQA10501-DQB10301 (3.3); H-2-IAb (42). |
|  | HEKGYAIPAINVTSS | 0% | HLA-DRB10101 (19.5); HLA-DRB10401 (436.1); HLA-DRB10404 (47.4); HLA-DRB10405 (381.2); HLA-DRB10701 (222.2); HLA-DRB10802 (175.9); HLA-DQA10101-DQB10501 (419); HLA-DQA10501-DQB10301 (27); H-2-IAb (47). |
| Glyceraldehyde-3-phosphate dehydrogenase (HCEG_09258) | NDNFGLTEGLMTTIH | 60% | HLA-DRB10101 (18.9); HLA-DRB10404 (236.2); HLA-DRB10405 (266.3); HLA-DRB10701 (40.8); HLA-DRB10901 (183.1); HLA-DPA10103-DPB10401 (177.5); HLA-DPA10103-DPB10201 (353); HLA-DPA10201-DPB10101 (80.2); HLA-DPA10103-HLA-DPB10301_DPB10401 (385.3); HLA-DPA10301-DPB10402 (73.4); HLA-DQA10102-DQB10602 (375.8); HLA-DQA10501-DQB10301(327.9). |
| Heat shock protein SSC1 (HCBG_08743) | KAKVDELQNASLTLF | 0% | HLA-DRB10101 (8.7); HLA-DRB10701 (52.7); HLA-DRB11302 (164.5); HLA-DRB40101 (496.6); HLA-DPA10103-DPB10201 (220.9); HLA-DPA10201-DPB10101 (100.4); HLA-DPA10301-DPB10402 (242.9). |
|  | VPSFARSSTYRLPTT | 0% | HLA-DRB10101 (8.6); HLA-DRB10401 (41); HLA-DRB10405 (74.3); HLA-DRB10701 (5.3); HLA-DRB10901 (24.3); HLA-DRB11101 (92.8); HLA-DRB11501 (404.5); HLA-DRB50101 (117.7). |
| Histone H15 (HCEG_07158) | KASASKAASSHASYR | 0% | HLA-DRB10101 (44.6); HLA-DRB50101 (205); HLA-DQA10102-DQB10602 (356.6); HLA-DQA10501-DQB10301 (8.6); H-2-IAb (343.6). |
|  | QSAFDTQFNRAVKTG | 0% | HLA-DRB10101 (7.1); HLA-DRB10401 (345.5); HLA-DRB11101 (141.4); HLA-DRB11302 (116.4); HLA-DRB50101 (209.2); HLA-DPA10103-DPB10201 (136.4); HLA-DPA10201-DPB10101 (474.6); HLA-DQA10501-DQB10301 (314.4). |
|  | KINITSQSAFDTQFN | 0% | HLA-DRB10101 (17.1); HLA-DRB10401 (279.4); HLA-DRB10701 (97.2); LA-DRB10802 (177.5); HLA-DRB11302 (377.9); HLA-DRB40101 (31.7); HLA-DRB50101 (282.6). |
|  | ALKKYVQANNKINIT | 0% | HLA-DRB10101 (24.8); HLA-DRB10401 (55.8); HLA-DRB10404 (109.1); HLA-DRB10405 (237.6); HLA-DRB10701 (33.9); HLA-DRB10802 (217); HLA-DRB10901 (239.7); HLA-DRB11101 (67.5); HLA-DRB11302 (14.5); HLA-DRB11501 (355.3); HLA-DRB50101 (34.3). |
| Homogentisate 1,2-dioxygenase (HCAG_05721) | PEKYTYLHGFGSYHE | 0% | HLA-DRB10101 (5.5); HLA-DRB10401 (114.7); HLA-DRB10404 (59.7); HLA-DRB10405 (36.9); HLA-DRB10701 (163.9); HLA-DRB10901 (68); HLA-DRB11101 (40.7); HLA-DRB11501 (24.6); HLA-DRB50101 (8.9); HLA-DPA10103-HLA-DPB10301_DPB10401 (415.7); HLA-DQA10501-DQB10301 (403.9). |
| HSP-60 (HCBG_08832) | ILGDIGILTNATVFT | 0% | HLA-DRB10101 (6.8); HLA-DRB10301 (50); HLA-DRB10401 (96.3); HLA-DRB10404 (27.7); HLA-DRB10405 (127.9); HLA-DRB10701 (243); HLA-DRB10802 (327.3); HLA-DRB10901 (498.8); HLA-DRB11101 (151.3); HLA-DRB11302 (82); HLA-DRB11501 (161.8); HLA-DQA10102-DQB10602 (326.1);  HLA-DQA10501-DQB10301 (87). |
|  | GTALLKAAANGLASV | 0% | HLA-DRB10101 (5.3); HLA-DRB10401 (136.6); HLA-DRB10404 (67.7), HLA-DRB10701 (7.4); HLA-DRB10802 (445.1); HLA-DRB10901 (43); HLA-DRB11101 (132.9); HLA-DRB11302 (20.4), HLA-DRB11501 (229.6); HLA-DRB40101 (464.4); HLA-DRB50101 (36); HLA-DQA10102-DQB10602 (92.9); HLA-DQA10501-DQB10301 (22.5); H-2-IAb (260.8). |
|  | SAKGEYVDMIASGIV | 41% | HLA-DRB10101 (5.9); HLA-DRB10401 (298.1); HLA-DRB10405 (229.8); HLA-DRB10701 (36.5); HLA-DRB10901 (65.7); HLA-DRB11501 (313.3); HLA-DRB50101 (88.3); HLA-DQA10102-DQB10602 (192.7); HLA-DQA10501-DQB10301 (198.8). |
|  | PTNFDQQLGVSIVKS | 50% | HLA-DRB10101 (5.6); HLA-DRB10405 (350.1); HLA-DRB10701 (181.4); HLA-DRB10901 (389.5); HLA-DRB11101 (401.4); HLA-DRB11302 (387.6); HLA-DRB11501 (468); HLA-DRB40101 (159); HLA-DRB50101 (86.6); HLA-DQA10102-DQB10602 (406.4); HLA-DQA10501-DQB10301 (148.2). |
|  | RASVLSSAPTRAPVS | 0% | HLA-DRB10101 (17); HLA-DRB10301 (104.5); LA-DRB10401 (87.6); HLA-DRB10404 (168.7); HLA-DRB10405 (320.2); HLA-DRB10701 (116.1); HLA-DRB10802 (175.4); HLA-DRB10901 (63.5); HLA-DRB11302 (333.9); HLA-DRB50101 (351.7); HLA-DQA10501-DQB10301 (29.2); H-2-IAb (63.7). |
| Hsp70-like protein (HCBG_07920) | ESYAYSLRNTLSDSK | 41.67% | HLA-DRB10101 (29.3); HLA-DRB10401 (55.3); HLA-DRB10404 (87.8); HLA-DRB10405 (37.8); HLA-DRB10701 (258.9); HLA-DRB10901 (361.1); HLA-DRB11101 (29.6); HLA-DRB11501 (264.4); HLA-DRB50101 (125.8); HLA-DPA10201-DPB10101 (269.5); HLA-DPA10103-HLA-DPB10301_DPB10401 (279); H-2-IAb (283.3); |
| Peroxidase (HCDG_01107) | STGYYLGLGAVLAGG | 0% | HLA-DRB10101 (4.1); HLA-DRB10401 (304.4); HLA-DRB10404 (196.7); HLA-DRB10405 (242.2); HLA-DRB10901 (42.7); HLA-DRB11101 (223.9); HLA-DRB11501 (326.7); HLA-DQA10102-DQB10602 (136.3); HLA-DQA10501-DQB10301 (9.4); H-2-IAb (364.9). |
|  | TKTLMMLPTDMALVK | 0% | HLA-DRB10101 (4.8); HLA-DRB10301 (453); HLA-DRB10401 (17.3); HLA-DRB10404 (5.7); HLA-DRB10405 (65.7); HLA-DRB10701 (394.7); HLA-DRB10901 (138.2); HLA-DRB11101 (365.6); HLA-DRB11501 (303.1); HLA-DRB40101 (38.9); HLA-DPA10201-DPB10101 (286.8); HLA-DPA10301-DPB10402 (259.7); HLA-DQA10102-DQB10602 (88.9). |
|  | EMVALSGAHSLGRAH | 0% | HLA-DRB10101 (7.3); HLA-DRB10401 (118.3); HLA-DRB10404 (126.5); HLA-DRB10701 (189.8); HLA-DRB10802 (149.3); HLA-DRB10901 (191); HLA-DRB11101 (158.5); HLA-DRB11501 (99.9); HLA-DRB50101 (13.4); HLA-DQA10102-DQB10602 (372.6); HLA-DQA10501-DQB10301 (37.6); H-2-IAb (494). |
|  | TRAFARAPLASGTSS | 0% | HLA-DRB10101 (15.1); HLA-DRB10401 (73); HLA-DRB10802 (119.2); HLA-DRB10901 (148); HLA-DRB11101 (173.4); HLA-DRB50101 (273.5); HLA-DQA10102-DQB10602 (409.1); HLA-DQA10501-DQB10301 (11.9); H-2-IAb (74). |
|  | DLWTLAGACAIQEMQ | 0% | HLA-DRB10101 (10.6); HLA-DRB10404 (136); HLA-DRB10701 (80.1); HLA-DRB10901 (74.3); HLA-DRB40101 (436.2); HLA-DQA10102-DQB10602 (90.2); HLA-DQA10301-DQB10302 (123); HLA-DQA10401-DQB10402 (38.3); HLA-DQA10501-DQB10201 (148.8); HLA-DQA10501-DQB10301 (38); H-2-IAb (326.4). |
| Ribosomal protein L14 (HCDG_01939) | VLVDGPAGQENK^*^ | 0% | HLA-DRB10301 (256.1). |
|  | HVLALSHATLTPFTIPK^*^ | 0% | HLA-DRB10101 (47.5); HLA-DRB10401 (89.1); HLA-DRB10404 (33.2); HLA-DRB10405 (209.6); HLA-DRB10701 (11.1); HLA-DRB10802 (400.9); HLA-DRB10901 (45.5); HLA-DRB11101 (257.8); HLA-DRB11501 (200.8); HLA-DRB40101 (89.7); HLA-DRB50101 (492.2); HLA-DPA10103-DPB10201 (246.5); HLA-DPA10201-DPB10101 (395.3); HLA-DQA10102-DQB10602 (238.9); HLA-DQA10501-DQB10301 (85.3). |
|  | FERFKVMRLKKQARY | 0% | HLA-DRB10101 (6.1); HLA-DRB10401 (270); HLA-DRB10404 (239.2); HLA-DRB10405 (408.8); HLA-DRB10701 (43.3); HLA-DRB10802 (397.2); HLA-DRB10901 (294.9); HLA-DRB11101 (5.9); HLA-DRB11501 (133.1); HLA-DRB40101 (40.3); HLA-DRB50101 (8.9); HLA-DPA10103-DPB10401 (34.2); HLA-DPA10103-DPB10201 (84.7); HLA-DPA10201-DPB10101 (40.6); HLA-DPA10201-DPB10501 (120); HLA-DPA10103-HLA-DPB10301_DPB10401 (83.5); HLA-DPA10301-DPB10402 (17.3). |
|  | TSSWKLVEVGRVVLI | 0% | HLA-DRB10101 (6.2); HLA-DRB10701 (56.4); HLA-DRB10901 (47); HLA-DRB11101 (487.8); HLA-DRB11501 (314.5); HLA-DRB50101 (179.8); HLA-DPA10201-DPB10101 (344.2); HLA-DQA10501-DQB10301 (66.7). |
|  | QARYEVQKAHAKIRA | 0% | HLA-DRB10101 (6.2); HLA-DRB10701 (122.8); HLA-DRB11101 (150.9); HLA-DRB40101 (429.4); HLA-DRB50101 (7.2); HLA-DQA10501-DQB10301 (56); H-2-IAd (338.2). |
| Sm protein (HCBG_00112) | TLGLTILRGTQVVSC | 0% | HLA-DRB10101 (4.5); HLA-DRB10404 (38.5); HLA-DRB10701 (53.3); HLA-DRB10802 (150.2); HLA-DRB10901 (295.7); HLA-DRB11101 (39.2); HLA-DRB11302 (219.8); HLA-DRB11501 (133.5); HLA-DRB40101 (289.5); HLA-DPA10201-DPB10101 (402.9); LA-DPA10301-DPB10402 (185.4); HLA-DQA10102-DQB10602 (122.4); HLA-DQA10501-DQB10301 (153.3). |
|  | RGLPVGLGGPAAGVG | 0% | HLA-DRB10101 (19.3); HLA-DQA10501-DQB10301 (6). |
| Mitochondrial thiamine pyrophosphate carrier 1 (HCDG_00690) | VSLWRGNTANVIRYF | 0% | HLA-DRB10101 (5.5); HLA-DRB10401 (222.4); HLA-DRB10404 (433.4); HLA-DRB10701 (60.3); HLA-DRB10901 (166); HLA-DRB11302 (6.1); HLA-DRB11501 (426); HLA-DRB30101 (39.2); HLA-DRB50101 (260.8); HLA-DQA10102-DQB10602 (274.2); HLA-DQA10501-DQB10301 (133); H-2-IAb (100). |
|  | VIRYFPTQALNFAFR | 0% | HLA-DRB10101 (5.7); HLA-DRB10401 (41.7); HLA-DRB10404 (63.8); HLA-DRB10405 (57.9); HLA-DRB10701 (18.5); HLA-DRB10802 (60.3); HLA-DRB10901 (25.8); HLA-DRB11101 (194.4); HLA-DRB11501 (13); HLA-DRB40101 (93.2); HLA-DRB50101 (98.3); HLA-DPA10103-DPB10401 (144.9); LA-DPA10103-DPB10201 (85); HLA-DPA10201-DPB10101 (31.1); HLA-DPA10201-DPB10501 (168.2); HLA-DPA10103-HLA-DPB10301_DPB10401 (27.4); HLA-DPA10301-DPB10402 (58.7); HLA-DQA10501-DQB10201 (271.2); H-2-IAb (217.9). |
|  | AGLYRGFGPSVLGIV | 0% | HLA-DRB10101 (6.8); HLA-DRB10401 (460.9); HLA-DRB10405 (276.7); HLA-DRB10701 (71.1); HLA-DRB10901 (10.8); HLA-DRB11101 (268.3); HLA-DRB11302 (197.3); HLA-DRB11501 (38.3); HLA-DRB50101 (253.1); HLA-DQA10501-DQB10301 (32.6); H-2-IAb (75.7). |
| Uncharacterized protein (HCDG_06427) | ATRPLRMQVTRPLLG | 0% | HLA-DRB10101 (6.1); HLA-DRB10401 (43.2); HLA-DRB10404 (150.3); HLA-DRB10405 (302.6); HLA-DRB10701 (27.8); HLA-DRB10802 (396.7); HLA-DRB10901 (80.1); HLA-DRB11101 (161.7); HLA-DRB11302 (194.4); HLA-DRB11501 (359.1); HLA-DRB30101 (100.5); HLA-DRB40101 (14); HLA-DRB50101 (73); HLA-DPA10103-DPB10401 (226.5); HLA-DPA10201-DPB10101 (423.6); HLA-DQA10102-DQB10602 (128.4); HLA-DQA10501-DQB10301 (391.3); H-2-IAd (223.9). |
|  | SIKKFRTDKTLRLSR | 0% | HLA-DRB10101 (7.2); HLA-DRB10301 (6.6); HLA-DRB10401 (56); HLA-DRB10404 (143.6); HLA-DRB10405 (153.8); HLA-DRB10701 (30.3); HLA-DRB10901 (100.6); HLA-DRB11101 (76.5); HLA-DRB11302 (78.6); HLA-DRB30101 (3.5); HLA-DRB50101 (100.7); HLA-DPA10103-DPB10401 (194.8); HLA-DPA10103-DPB10201 (400.1); HLA-DPA10201-DPB10101 (94.8); HLA-DPA10301-DPB10402 (56.6). |
| 4-Hydroxyphenylpyruvate dioxygenase (HCBG_03027) | IFTEFSALKSVVMAS | 46.67% | HLA-DRB10101 (5); HLA-DRB10401 (30.4); HLA-DRB10404 (39); HLA-DRB10405 (65.6); HLA-DRB10701 (15.7); HLA-DRB10901 (67.6); HLA-DRB11101 (33.8); HLA-DRB11501 (128.6); HLA-DRB50101 (19.4); HLA-DPA10103-DPB10401 (248.1); HLA-DPA10201-DPB10101 (56); HLA-DPA10103-HLA-DPB10301_DPB10401 (460.5); HLA-DPA10301-DPB10402 (37.6); HLA-DQA10102-DQB10602 (135.6). |
| 6-Phosphogluconate dehydrogenase (HCBG_03027) | RTKYLAGKNIRFVGS | 0% | HLA-DRB10101 (5.1); HLA-DRB10401 (19.1); HLA-DRB10404 (293.8); HLA-DRB10405 (294.9); HLA-DRB10701 (58.2); HLA-DRB10901 (128.9); HLA-DRB11101 (82.6); HLA-DRB11302 (234.7); HLA-DRB11501 (76.9); HLA-DRB50101 (40.1); HLA-DPA10201-DPB10501 (258); HLA-DQA10501-DQB10301 (415). |
| Ribosomal protein S23  (HCEG_08043) | LTYKKRLLGTAFKSS | 61% | HLA-DRB10101 (19.1); HLA-DRB10404 (321.4); HLA-DRB10405 (143.9); HLA-DRB10802 (270.6); HLA-DRB10901 (205.5); HLA-DRB11101 (34); HLA-DRB11501 (458.2); HLA-DRB50101 (14); HLA-DPA10103-DPB10401 (62.2); HLA-DPA10103-DPB10201 (9.4); HLA-DPA10201-DPB1010 (89.2); HLA-DPA10201-DPB10501 (57.9); HLA-DPA10103-HLA-DPB10301_DPB10401 (99.1); HLA-DPA10301-DPB10402 (352.9). |
| 40S ribosomal protein S15 (HCBG_01774) | LRDVVHARARRRFNR | 53% | HLA-DRB10101 (8.5); HLA-DRB10301 (265.1); HLA-DRB10701 (79.1); HLA-DRB10901 (374); HLA-DRB11101 (22); HLA-DRB11501 (227.4); HLA-DRB50101 (9.8); HLA-DQA10501-DQB10301 (247.3). |
| 60S ribosomal protein (HCEG_04281) | IIVHPLNTESAMK^*^ | 0% | HLA-DRB10101 (373.8); HLA-DRB10401 (483.4); HLA-DRB10405 (405.5); HLA-DRB10802 (235.9). |
|  | KAVLKGVHSHKVRKI | 0% | HLA-DRB10101 (7); HLA-DRB10401 (408.1); HLA-DRB10701 (402.1); HLA-DRB10802 (191.2); HLA-DRB10901 (192.9); HLA-DRB11101 (87.4); HLA-DRB11302 (482.5); HLA-DRB11501 (166); HLA-DRB50101 (48.4); HLA-DQA10501-DQB10301 (468.2). |
|  | STTFHRPKTLQLSRS | 0% | HLA-DRB10101 (17.4); HLA-DRB10701 (43); HLA-DRB10901 (234.3); HLA-DRB11101 (253.5); HLA-DRB50101 (220.2). |
|  | KPSAKAGAAAKAVLK | 0% | HLA-DRB10101 (24.2); HLA-DRB50101 (47.9); HLA-DQA10102-DQB10602 (282); HLA-DQA10501-DQB10301 (5.5); H-2-IAd (281.9); H-2-IAb (174.9). |
| 60S ribosomal protein L2  (HCDG_08240) | GQFIYAGKNATLTVG | 0% | HLA-DRB10101 (5); HLA-DRB10401 (48.6); HLA-DRB10404 (262.3); HLA-DRB10405 (99.3); HLA-DRB10701 (118.4); HLA-DRB10802 (182); HLA-DRB10901 (41.5); HLA-DRB11101 (29.8); HLA-DRB11302 (28.5); HLA-DRB11501 (79.2); HLA-DRB50101 (36.1); HLA-DQA10501-DQB10301 (15.3); H-2-IAb (92.3). |
|  | VETFIANEGMYTGQF | 0% | HLA-DRB10101 (6.3); HLA-DRB10401 (180.8); HLA-DRB10404 (192.1); HLA-DRB10405 (468.6); HLA-DRB10901 (409.9); HLA-DRB11302 (70.2); HLA-DRB11501 (130.2); HLA-DRB30101 (23.3); HLA-DPA10103-DPB10201 (337.5); HLA-DPA10201-DPB10101 (453.2); HLA-DQA10102-DQB10602 (170.9); HLA-DQA10501-DQB10301 (496.1). |
| 60S ribosomal protein L27  (HCEG_00238) | VVIIQPYDAGSK^*^ | 0% | HLA-DRB10101 (21.2); HLA-DRB10404 (268.3); HLA-DRB11501 (283.2); HLA-DRB50101 (185.9). |
|  | MKFMKVGRVAIITRG | 50% | HLA-DRB10101 (12.7); HLA-DRB10404 (258.9); HLA-DRB10701 (88.3); HLA-DRB10802 (294); HLA-DRB10901 (341.4); HLA-DRB11101 (30.3); HLA-DRB11302 (223.9); HLA-DRB11501 (160.2); HLA-DRB40101 (221.1); HLA-DRB50101 (223.3); HLA-DPA10301-DPB10402 (315.8); HLA-DQA10102-DQB10602 (375.8); HLA-DQA10501-DQB10301 (28.3); H-2-IAd (70.1). |
|  | TLELEGLKGAITNDT | 0% | HLA-DRB10101 (7.3); HLA-DRB10405 (338.4); HLA-DQA10501-DQB10301 (36.9). |

^a^ Protein: Protein name and Access number to Uniprot database.

^b^ HS (%) - Human Similarity: Percentage of similarity of the peptide sequence predicted as T cell epitope with the human protein sequence.

^c^ Allele (IC50 nM): The values shown indicate strong binders (SB) when IC50 <50 nm and intermediate binders (IB) when IC50 <500 nm. Weak binders (WB) were not considered.

^*^ Sequences of peptides obtained through the method of immunoprecipitation that were naturally presented by Mφ and DCs of mice.

**Supplementary Table 5.** MHC-I and II epitopes restricted to *H. capsulatum* predicted immunogenic.

| **Protein** | **MHC-I / Peptide sequence** | **MHC-II / Peptide sequence** | **Accession number^a^** |
| --- | --- | --- | --- |
| **Aha1_N** | DVVKRNWEGY | GEYIKLESPTSITQK  RKELAKLAPALIAEH | HCEG_02400 |
| **Aldehyde dehydrogenase** | AIEIANNTSY  TPTNRGRMLI | LDNYTQIKSVRVRLG | HCAG_08367 |
| **ATP synthase** | SPTEISSAL | - | HCBG_01891 |
| **ATP-dependent molecular chaperone HSC82** | FISYPIYLHV  KLLRFNSTK | FAERIHKLVSLGLNI | HCAG_04686 |
| **Citrate synthase** | ESFHDLLRLY | - | HCEG_09064 |
| **Elongation factor 1-gamma** | SELVPTLGGW | GGFFTRLEASRKYVF  IAIYLASQNEKTTLL | HCBG_08684 |
| **Enolase** | TEAIEQAGY  KPYVLPVPF  YVLPVPFQNV | VTNPLRIKKAIELKA  EFMIVPSTAPSFSEA  AFQEFMIVPSTAPSF | HCEG_02034 |
| **Fructose-biphosphato aldolase** | RLFEHAHEK  KYFDPRVWI  AEFKEAISY | ERYFKLHGEPLFSSH  DMQYAYLSGVRDYVL  YAIYKTLSAISPYFS | HCBG_06745 |
| **Glyceraldehyde-3-phosphate dehydrogenase** | GELKGILGY  ETHYAAYMLK | - | HCEG_09258 |
| **Heat shock protein SSC1** | GEVTDVLLL | VPSFARSSTYRLPTT | HCBG_08743 |
| **Histone H15** | MIKDAIINL  RKAPVAPAV | ALKKYVQANNKINIT | HCEG_07158 |
| **Homogentisate 1,2-dioxygenase** | SEAIDGALPV  YTYLHGFGSY | - | HCAG_05721 |
| **HSP70** | KSEIDKTVQW | - | HCBG_07920 |
| **HSP-60** | FENLGARLL  LTDEHASDF  LEKATADML  IADPTTSDY | GTALLKAAANGLASV  ILGDIGILTNATVFT | HCBG_08832 |
| **Peroxidase** | FTNEFFRLLV  REFSDAFVKL  MMLPTDMALV | EMVALSGAHSLGRAH  TKTLMMLPTDMALVK | HCDG_01107 |
| **Ribosomal protein L14** | LSHATLTPF  RVVLIRSGPY | - | HCDG_01939 |
| **Sm protein** | RPAGRGLPV | - | HCBG_00112 |
| **Mitochondrial thiamine pyrophosphate carrier 1** | KSDGIAGLY  RYFPTQALNF | VSLWRGNTANVIRYF  AGLYRGFGPSVLGIV  VIRYFPTQALNFAFR | HCDG_00690 |
| **Uncharacterized protein** | ALGAGIYSS  LPLGAVLCV | ATRPLRMQVTRPLLG  SIKKFRTDKTLRLSR | HCDG_06427 |
| **4-Hydroxyphenylpyruvate dioxygenase** | EYYEKVLGF | IFTEFSALKSVVMAS | HCBG_03027 |
| **6-Phosphogluconate dehydrogenase** | FIDNLEQALY  VFLKDITNAY | RTKYLAGKNIRFVGS | HCEG_08718 |
| **40S ribosomal protein S15** | VPEMIGSII | - | HCBG_01774 |
| **60S ribosomal protein** | RTSTTFHRPK  HPLNTESAM | - | HCEG_04281 |
| **60S ribosomal protein L2** | ETFIANEGMY  KYKDVVETF  FIYAGKNATL | GQFIYAGKNATLTVG | HCDG_08240 |
| **60S ribosomal protein L27** | ITRGRYAGK  KAHPFPYAL | MKFMKVGRVAIITRG | HCEG_00238 |

^a^ Access number to the Uniprot database.

**Supplementary Table 6.** IFN-γ inducing peptides in a model for prediction of IFN-γ versus non-IFN-γ.

| **Protein** | **Sequence** | **Accession number^a^** |
| --- | --- | --- |
| **Aha1_N** | RKELAKLAPALIAEH | HCEG_02400 |
| **Aldehyde dehydrogenase** | LDNYTQIKSVRVRLG | HCAG_08367 |
| **ATP-dependent molecular chaperone HSC82** | KKTFEISPKSPIIQE  HEDAQNRPALAKLLR | HCAG_04686 |
| **Elongation factor 1-gamma** | GGFFTRLEASRKYVF | HCBG_08684 |
| **Enolase** | TADCQIVGDDITVTNPLR  AFQEFMIVPSTAPSF VTNPLRIKKAIELKA | HCEG_02034 |
| **Fructose-biphosphato aldolase** | DYLLTAVGNPEGDDKPNKK  ERYFKLHGEPLFSSH  SPLILQVSQGGAAFFAGK | HCBG_06745 |
| **Glyceraldehyde-3-phosphate dehydrogenase** | NDNFGLTEGLMTTIH | HCEG_09258 |
| **Heat shock protein SSC1** | KAKVDELQNASLTLF | HCBG_08743 |
| **Histone H15** | QSAFDTQFNRAVKTG | HCEG_07158 |
| **Peroxidase** | STGYYLGLGAVLAGG  EMVALSGAHSLGRAH | HCDG_01107 |
| **Ribosomal protein L14** | TSSWKLVEVGRVVLI  QARYEVQKAHAKIRA  HVLALSHATLTPFTIPK | HCDG_01939 |
| **Sm protein** | RGLPVGLGGPAAGVG | HCBG_00112 |
| **Mitochondrial thiamine pyrophosphate carrier 1** | VSLWRGNTANVIRYF  VIRYFPTQALNFAFR  AGLYRGFGPSVLGIV | HCDG_00690 |
| **Uncharacterized protein** | ATRPLRMQVTRPLLG SIKKFRTDKTLRLSR | HCDG_06427 |
| **60S ribosomal protein** | STTFHRPKTLQLSRS | HCEG_04281 |
| **60S ribosomal protein L2** | GQFIYAGKNATLTVG | HCDG_08240 |
| **60S ribosomal protein L27** | TLELEGLKGAITNDT | HCEG_00238 |

^a^ Access number to Uniprot database.
